# Multi‐Layered Triboelectric Nanogenerators with Controllable Multiple Spikes for Low‐Power Artificial Synaptic Devices

**DOI:** 10.1002/advs.202304598

**Published:** 2023-10-27

**Authors:** Yong‐Jin Park, Yun Goo Ro, Young‐Eun Shin, Cheolhong Park, Sangyun Na, Yoojin Chang, Hyunhyub Ko

**Affiliations:** ^1^ School of Energy and Chemical Engineering Ulsan National Institute of Science and Technology (UNIST) 50, UNIST‐gil Ulsan 44919 Republic of Korea

**Keywords:** artificial synaptic devices, human‐machine interface, transistors, triboelectric nanogenerators

## Abstract

In the domains of wearable electronics, robotics, and the Internet of Things, there is a demand for devices with low power consumption and the capability of multiplex sensing, memory, and learning. Triboelectric nanogenerators (TENGs) offer remarkable versatility in this regard, particularly when integrated with synaptic transistors that mimic biological synapses. However, conventional TENGs, generating only two spikes per cycle, have limitations when used in synaptic devices requiring repetitive high‐frequency gating signals to perform various synaptic plasticity functions. Herein, a multi‐layered micropatterned TENG (M‐TENG) consisting of a polydimethylsiloxane (PDMS) film and a composite film that includes 1H,1H,2H,2H‐perfluorooctyltrichlorosilane/BaTiO_3_/PDMS are proposed. The M‐TENG generates multiple spikes from a single touch by utilizing separate triboelectric charges at the multiple friction layers, along with a contact/separation delay achieved by distinct spacers between layers. This configuration allows the maximum triboelectric output charge of M‐TENG to reach up to 7.52 nC, compared to 3.69 nC for a single‐layered TENG. Furthermore, by integrating M‐TENGs with an organic electrochemical transistor, the spike number multiplication property of M‐TENGs is leveraged to demonstrate an artificial synaptic device with low energy consumption. As a proof‐of‐concept application, a robotic hand is operated through continuous memory training under repeated stimulations, successfully emulating long‐term plasticity.

## Graphical abstract

Three‐layered triboelectric nanogenerator (3‐TENG) generates multiple spikes from a single touch and its integration with an organic electrochemical transistor (OECT) to accomplish a highly efficient artificial synaptic device. These multiple spikes not only enhance the triboelectric performance of the TENG but can also be utilized to successfully emulate neural functions by delivering high‐frequency gate voltage to the OECT.

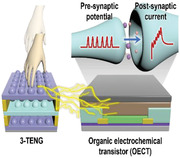

## 1 Introduction

A triboelectric nanogenerator (TENG) is a device that generates electrical energy from mechanical contacts, making it applicable to various sensors, such as electronic skins,^[^ ^1^, ^2^, ^3^ ^]^ body motion sensors,^[^ ^4^, ^5^, ^6^ ^]^ robotic tactile sensors,^[^ ^7^, ^8^, ^9^ ^]^ and energy harvesting devices utilizing vibration,^[^ ^10^, ^11^ ^]^ wind,^[^ ^12^, ^13^, ^14^ ^]^ wave energy,^[^ ^15^, ^16^, ^17^ ^]^ and more. With the growing interest in developing human‐machine interfaces (HMIs), there is a need for devices that consume low power and have multiplex sensing, memory, and learning functions. TENGs are highly versatile in this regard, especially when combined with synaptic transistors, which mimic biological synapses to realize both memory and learning functions. Combining the sensing and energy harvesting capabilities of TENGs with the memory and learning functions of synaptic devices allows for intelligent sensing in neuromorphic devices with low power consumption. When integrated with synaptic transistors, TENGs can provide spike‐like impulses to the gate of transistors, which is analogous to action potential propagation in biological systems.^[^ ^18^, ^19^ ^]^ Additionally, self‐powered triboelectric potential gating can replace the need for an external power supply to operate synaptic transistors, thus reducing energy consumption. This efficiency is crucial for neuromorphic systems, which are more energy‐efficient compared to the conventional von Neumann computing architecture.^[^ ^20^ ^]^ One of the key aspects of emulating neural functions is replicating synaptic plasticity, which includes short‐term plasticity (STP) and long‐term plasticity (LTP). Synaptic transistors emulate this function by correlating channel conductance with synaptic weight, representing the strength of connection between pre‐synaptic and post‐synaptic neurons.^[^ ^21^ ^]^ STP, which involves rapid changes in synaptic weight that occur over a few milliseconds to seconds, is responsible for perceptual and cognitive functions.^[^ ^22^ ^]^ LTP, on the other hand, entails changes in synaptic weight that last for several hours or longer, making it fundamental to learning and memory functions.^[^ ^23^ ^]^ Therefore, achieving LTP is essential for the development of neuromorphic computing systems. The transition from STP to LTP in neural networks is achieved by strengthening neural connections through repetitive high‐frequency stimuli.^[^ ^24^ ^]^ Similarly, for tribotronic synaptic transistors to exhibit LTP behavior, it is desirable for TENGs to supply high‐frequency gate voltages to the transistors. Previous efforts to integrate TENGs with synaptic transistors mainly relied on input pressures higher than 10 Hz,^[^ ^25^, ^26^, ^27^ ^]^ which limits the types of external stimuli that can be used.

To harness TENGs in various applications, significant efforts have been devoted to enhancing their triboelectric output performance. The majority of earlier studies have concentrated on selecting materials with a significant triboelectric polarity difference in the triboelectric series,^[^ ^28^ ^]^ as well as on structural designs such as modifying the surface of triboelectric layers with micro‐ and nanostructures and fine‐tuning the overall device structures.^[^ ^29^, ^30^, ^31^, ^32^ ^]^ Recently, multi‐layered structures with a larger surface area have gained popularity for their potential to boost triboelectric output performance. For example, multi‐layered TENGs based on mesoporous polydimethylsiloxane (PDMS)/Au‐Al/Al films,^[^ ^33^ ^]^ wavy Cu‐Kapton‐Cu films,^[^ ^34^ ^]^ corona charging‐treated polytetrafluoroethylene (PTFE) electret films,^[^ ^35^ ^]^ and multi‐gap structured polyvinylidene fluoride and nylon films have demonstrated an increase in triboelectric output performance proportional to the number of layers.^[^ ^36^ ^]^ Previous studies on multi‐layered structures have made significant progress in enhancing energy harvesting performance by utilizing large surface area multi‐layers. While these multi‐layered structures have achieved significant advancements in energy harvesting by leveraging large surface areas, no studies have been reported on employing the multiple triboelectric spikes generated by these multi‐layered structures from a single touch as a source of repetitive, high‐frequency gate voltage for achieving LTP in synaptic devices (Table S1, Supporting Information).

In this study, we introduce a multi‐layered TENG (M‐TENG) designed to produce multiple triboelectric spikes with a single touch. Additionally, we demonstrate its integration with an organic electrochemical transistor (OECT) to create an artificial synaptic device. This device boasts lower power consumption and is capable of emulating the synaptic plasticity and learning behavior of the brain. The M‐TENG employs bifacial micropatterned friction multi‐layers made of PDMS composite films infused with high‐k BaTiO_3_ (BTO) nanoparticles. These films are treated with 1H,1H,2H,2H‐perfluorooctyltrichlorosilane (FOTS) self‐assembled monolayers (SAMs) to enhance the triboelectric polarity difference between the friction layers. Unlike conventional TENGs, which generate only two spikes per contact‐separation cycle, our M‐TENG produces multiple distinct spikes, the number of which depends on the number of layers and the pressure applied during a single touch. The generation of multiple spikes in our M‐TENG is achieved as each friction layer generates its own triboelectric signal through the induced charges at the contact surfaces. Additionally, the spacers between layers create a contact/separation delay, allowing for the signals to be distinct. As the number of friction layers increases, both the triboelectric output performance and the number of spikes increase. Furthermore, the number of spikes can be modulated by adjusting the pressure applied, resulting in sequential contacts of the friction layers from top to bottom as pressure increases. Notably, micropatterns facilitate low adhesion and clear separation between friction layers during contact and separation, thereby preventing damping effects. The M‐TENG's ability to generate multiple spike signals makes it particularly well‐suited for use in artificial synaptic devices with low energy consumption, especially when paired with an OECT. In this configuration, the M‐TENG acts as a mechanoreceptor, supplying the pre‐synaptic potential through mechanical stimulus. This pre‐synaptic potential is then used to regulate the gate of the OECT (representing the pre‐synaptic neuron), which in turn modulates the drain current in the OECT channel (acting as the post‐synaptic neuron). This modulation represents the excitatory post‐synaptic current (EPSC), and the high‐frequency output signals from the M‐TENG can effectively enhance the EPSC. The EPSC can be facilitated with repeated stimulations, inducing a transition from STP to LTP with a high synaptic weight. As a proof‐of‐concept application in HMIs, we demonstrate that an M‐TENG coupled with an OECT can be used to control a robotic hand. This system is capable of simulating memory training, also referred to as associative learning, in response to external stimuli.

## 2 Results and Discussion

### 2.1 M‐TENGs Generating Multiple Spikes with a Single Touch

**Figure 1:**
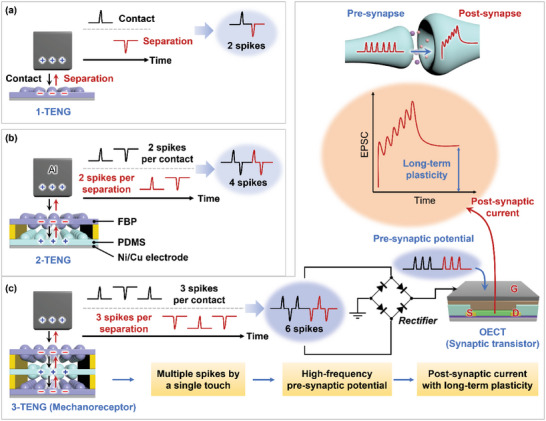
Schematic illustration of multi‐layered micropatterned triboelectric nanogenerators (M‐TENGs) with multiple spikes triggered by a single touch and their application in artificial synaptic devices. a) Conventional 1‐TENG, b) 2‐TENG, and c) 3‐TENG devices generate two, four, and six spikes, respectively. 3‐TENG was used to provide high‐frequency pre‐synaptic potential to achieve post‐synaptic current with long‐term plasticity in an artificial synapse, which consists of a 3‐TENG, a full‐wave bridge rectifier, and an OECT.

**Figure** **1** illustrates the concept, structure, and application of the M‐TENGs. To fabricate M‐TENGs, we used Al, PDMS, and FOTS/BTO/PDMS (FBP) as friction layers. Because Al exhibits a relatively high tribo‐positive property compared to the tribo‐negative property of PDMS film, we used Al tape as both a tribo‐positive material and an electrode. M‐TENGs consist of alternately stacked PDMS and FBP layers, separated by spacers. These spacers maintain a certain distance between two friction layers and create an instantaneous contact/separation delay, resulting in distinct multiple output spikes. In contrast to the conventional principle of generating a large number of output spikes by increasing the intrinsic working frequency of the device,^[^ ^37^ ^]^ M‐TENGs are more efficient in terms of triboelectric generation because they produce multiple output spikes with a single touch (stimulus). This advantage of M‐TENGs can be harnessed to develop an efficient artificial synaptic device that facilitates synaptic weight with low‐frequency mechanical stimuli.

In the contact‐separation mode of a conventional single‐layered TENG, referred to as 1‐TENG in this study, two spike signals are generated: one during the contact process and another, with opposite polarity, during the separation process between the Al tape and the PDMS film (Figure 1a). These two bidirectional spikes are attributed to the effects of contact electrification and electrostatic induction through the contact‐separation mode. On the other hand, 2‐TENG, which consists of FBP as a tribo‐negative layer and PDMS film as a tribo‐positive layer, generates two spike signals during the contact process and two additional spikes of opposite polarity during the separation process (Figure 1b). As such, 2‐TENG produces twice as many spikes as 1‐TENG with a single touch. The generation of these additional spikes in 2‐TENG can be attributed to the sequential difference in triboelectric properties among Al, FBP, and PDMS film, as well as to the spacers between layers that induce contact/separation delays. The detailed working mechanism of M‐TENG is explained in Section 2.2.

The dual‐layered TENG (2‐TENG) is composed of two stacked dielectric films with different triboelectric properties between electrodes. To optimize its triboelectric output performance, we first modulated the pitch of the micropatterns to 12, 15, and 17 µm (Figure S1a–c, Supporting Information) to evaluate the triboelectric performances (short‐circuit current density (*J*_sc_) and open‐cirucuit voltage (*V*_oc_)) as shown in Figure S1d,e, Supporting Information. The triboelectric performance of the micropatterned PDMS (M‐PDMS) with a pitch of 15 µm was similar to that of the 12 µm pitch. In contrast, the M‐PDMS with a pitch of 17 µm exhibited higher triboelectric performances. However, the M‐PDMS with a pitch of 17 µm accompanied by a damping effect due to its increased adhesive strength. This phenomenon will be discussed in detail later. Considering these results, the micropatterned PDMS with a pitch of 12 µm was ultimately adopted. Next, we incorporated BTO nanoparticle (NP) fillers into PDMS to fabricate a BTO/PDMS (BP) composite film and then coated the surface with FOTS to produce a FOTS/BTO/PDMS (FBP) composite film (Figure S2, Supporting Information). The ferroelectric BTO fillers enhance the Maxwell–Wagner–Sillars (MWS) interfacial polarization effect and the amount of accumulated charges, which, in turn, boost the triboelectric output performances.^[^ ^38^, ^39^ ^]^ The MWS interfacial polarization effect occurs at heterogeneous dielectric composites, such as a PDMS matrix with BTO ferroelectric fillers, where the oscillating dipoles in the BTO nanoparticles are aligned when the electrical field is generated during the working process of TENG. The aligned dipoles trigger the interfacial polarization at the PDMS matrix and BTO interface, which leads to the significant accumulation of charges at the interfaces or boundaries of the ferroelectric particles (Figure S3, Supporting Information).^[^ ^40^ ^]^ This effect improves both the real part of permittivity, indicative of the polarization capability of the dielectric material and the charge density, resulting in a high *J*_sc_. In 2‐TENG, the additional FBP layer becomes more triboelectrically negative in the triboelectric series as BTO NPs are added and the surface is coated with FOTS, maximizing the relative triboelectric polarity difference. While varying the BTO weight concentrations (0, 1, 5, and 10 wt%), we found that *J*_sc_ and *V*_oc_ of BP composite films were highest at 5 wt% BTO (Figure S2a,b, Supporting Information). The inclusion of BTO NPs induces effective interfacial polarization in the polymer composite, allowing for the accumulation of more interfacial charges,^[^ ^41^, ^42^ ^]^ leading to increasing triboelectric performance, in accordance with the MWS interfacial polarization effect. However, at higher BTO concentrations (10 wt%), the triboelectric performance deteriorated due to the aggregation of BTO NPs, which resulted in decreased interfacial polarization. The enhancement of the triboelectric performance through the MWS interfacial polarization effect was also evaluated using another nanoparticle material, namely, TiO_2_ (Figure S4, Supporting Information). Although the TiO_2_ NPs‐embedded PDMS also showed the MWS effect due to the trapped charge carriers at the interfaces between TiO_2_ fillers and the PDMS matrix,^[^ ^43^ ^]^ the performance enhancement was not as substantial as that of the BP composite (5 wt%). This difference can be attributed to the fact that the relative permittivity of BTO is much higher than that of TiO_2_.^[^ ^38^ ^]^ The FOTS molecule, rich in fluorine, exhibits a strong electron affinity, contributing to a high triboelectric output performance.^[^ ^44^ ^]^ As a result, the *J*_sc_ and *V*_oc_ of the BP film coated with FOTS were higher than those without FOTS coating (Figure S2c,d, Supporting Information).

The above results indicate that Al, PDMS, and FBP are properly positioned in the triboelectric series to generate high triboelectric signals (Figure S2e, Supporting Information). The FOTS coating on the BP was analyzed using X‐ray photoelectron spectroscopy (XPS), and the representative C and F spectrum is shown in Figure S5, Supporting Information. In the C 1s region, C–C, C–O, CF_2_, and CF_3_ bonds in FBP were observed at 284.1, 285.6, 291.4, and 293.6 eV, respectively (Figure S5a, Supporting Information).^[^ ^45^, ^46^ ^]^ In addition, in the F 1s region, organic F containing CF_3_, CF, and CF_2_ on the FBP surface were detected at 687.9, 688.6, and 689.4 eV, respectively (Figure S5b, Supporting Information). However, no peaks corresponding to PDMS were found in the F 1s region. The XPS results indicate the formation of chemically homogeneous FOTS on PDMS. X‐ray diffraction (XRD) results (Figure S6, Supporting Information) confirm that the crystalline BTO NPs were evenly distributed in the amorphous PDMS matrix without alternation of the chemical structure, exhibiting the amorphous nature of PDMS (a broad peak at 11.8°),^[^ ^47^ ^]^ and the main crystalline peaks of BTO NPs.^[^ ^42^ ^]^

PDMS is an ideal friction material due to its excellent mechanical and chemical properties, which facilitate the reversible contact‐separation process against the counterpart layer.^[^ ^38^ ^]^ However, due to the soft nature of PDMS, partial adhesion occurs between the friction layers during contact‐separation cycles, preventing complete separation and resulting in indistinct contact/separation peaks during TENG operation. To address this issue, a M‐PDMS structure was employed to reduce adhesive strength with the opposing layer. The morphology of micropatterns was examined using a field emission scanning electron microscope (FE‐SEM) (Figure S7, Supporting Information). The cross‐sectional FE‐SEM images show that BTO NPs are homogeneously distributed within the PDMS matrix without aggregation. In contrast, planar PDMS films without micropatterns tend to have significant adhesion issues, accompanied by damping in the friction layer. As shown in Figures S1 and S8, Supporting Information, adhesion tests were conducted with M‐PDMS with different pitches (10, 12, and 17 µm) and various triboelectric pair materials, including Al–planar PDMS, Al–M‐PDMS, planar PDMS–planar PDMS and M‐PDMS–M‐PDMS. During the separation process of 2‐TENG, the planar failed to separate instantly (within 0.3 s) due to the adhesion at the Al–planar BP and BP–PDMS interfaces (Figure S8a‐i,ii, Supporting Information). Instead, they separated later (after 0.6 s) accompanied by an unintended damping effect (Figure S8a‐iii, Supporting Information). This adhesion issue in planar layers introduces a damping effect, resulting in unclear *J*_sc_ signals (Figure S8b, Supporting Information), underlining the importance of microstructural surface modification of the friction layers. The adhesive strengths of each M‐PDMS with different pitches (12, 15, and 17 µm) were measured against Al (Figure S1f, Supporting Information). The adhesive strengths of M‐PDMSs with pitches of 12 and 15 µm were ≈0 kPa, whereas that of the M‐PDMS with a pitch of 17 µm was 3.8 kPa. Furthermore, while the adhesive strengths between Al–planar PDMS and planar PDMS–planar PDMS layers were found to be 6.9 and 3.3 kPa, respectively, those between Al–M‐PDMS and M‐PDMS–M‐PDMS layers were ≈0 kPa (Figure S8c, Supporting Information).

A triple‐layered TENG (3‐TENG) was fabricated by adding an additional PDMS layer to the 2‐TENG, resulting in the generation of three spike signals during the contact process and another three during the separation process (Figure 1c). Therefore, 3‐TENG produces a total of six spikes, which is three times higher than that of 1‐TENG. The increased number of spikes produced by the 3‐TENG not only enhances triboelectric output performance compared to 1‐TENG under the same mechanical stimuli in terms of pressure and frequency but also offers a higher frequency of triboelectric signals that can effectively induce long‐term plasticity in artificial synaptic devices. When the 3‐TENG is integrated into an artificial synaptic device, the six spikes generated by the 3‐TENG (acting as a mechanoreceptor) with a single touch are directed through a full‐wave bridge rectifier, converting all bidirectional spikes into unidirectional ones. These spikes emulate the pre‐synaptic potentials that occur when human skin perceives external mechanical stimuli. This spike potential leads to a dense accumulation of high‐frequency stimulation, which is transferred to the gate of the OECT (representing a pre‐synaptic neuron). As a result, the drain current of the OECT, which corresponds to EPSC in the post‐synaptic neuron, is modulated. Upon repeated stimulation, the EPSC is amplified, facilitating an efficient transition from STP to LTP with increased synaptic weight. Therefore, M‐TENGs can be effectively deployed in neuromorphic applications, as their high‐frequency output signals readily enhance EPSC.

### 2.2 Working Mechanism of M‐TENG

**Figure 2:**
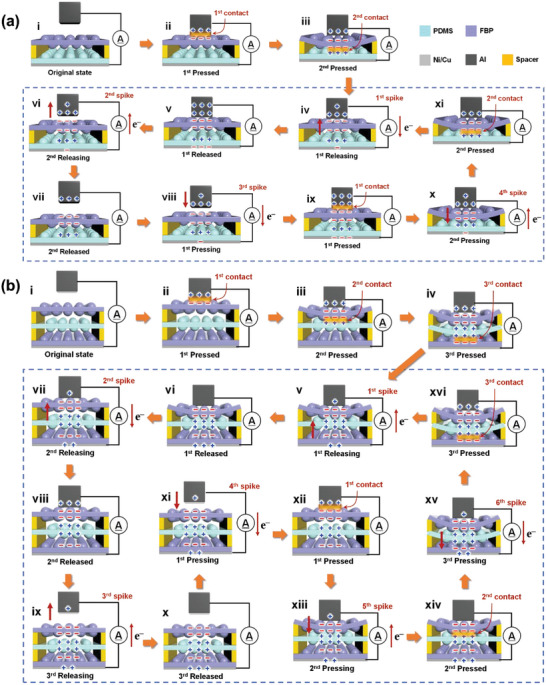
Working mechanisms of multiple spike generation in a) 2‐TENG and b) 3‐TENG. As pressure increases or decreases, the friction layers sequentially make contact from top to bottom or separate from bottom to top, inducing different electron flow behaviors in each stage of the contact and separation processes. These different electron flow behaviors result in a) four bidirectional spikes for 2‐TENG and b) six bidirectional spikes for 3‐TENG.

The working mechanism of the M‐TENG is shown in **Figure** **2**. For the 2‐TENG, the working mechanism can be divided into four stages of electron flow (Figure 2a). When the tribo‐positive Al contacts with the tribo‐negative FBP, Al and the upper surface of FBP become oppositely charged (triboelectrification, stage ii). In stage iii, when the Al layer continues to press down and forces the FBP layer to make contact with the PDMS layer, the lower surface of FBP and the surface of PDMS become oppositely charged. When the FBP and PDMS layers are separted, electrons flow from the Al to the Ni/Cu electrodes through the external circuit to compensate for the potential difference (electrostatic induction), resulting in the generation of the 1^st^ spike (stages v and vi). In stage vii, electrons flow back from the Ni/Cu to the Al electrodes to compensate for the opposite charges, leading to the generation of the 2^nd^ spike. Similarly, in stages viii and x, opposing electron migrations occur when the each layer is pressed, generating the 3^rd^ and 4^th^ spikes. These four different electron flow behaviors result in four different bidirectional spikes in *J*_sc_.

The working mechanism of the 3‐TENG is similar to that of the 2‐TENG, but with six stages of electron flow behaviors, resulting in six bidirectional spikes in *J*_sc_ (Figure 2b). During stage ii, the Al layer contacts with the first upper surface of the FBP layer, inducing opposite charges on the two surfaces. In stage iii, as the FBP layer is pushed down to make contact with the PDMS layer, the negative charges on the upper surface of the FBP layer are maintained. Simultaneously, since PDMS is tribo‐positive material against FBP additional negative and positive charges are newly generated at the lower surface of FBP and the upper surface of the PDMS layers, respectively. In stage iv, when all the friction layers are fully pressed and in contact with each other, the second FBP layer and the lower surface of the PDMS layer become negatively and positively charged, respectively. During the separation process, all the friction layers are completely separated, causing electron flows and generating three spikes (1^st^, 2^nd^, and 3^rd^ spikes in stages v, vii, and ix, respectively). The 4^th^, 5^th^, and 6^th^ spikes are generated during stages xi, xiii, and xv when each layer is pressed, causing opposite electron flows. At each stage, the net charge in the TENG system becomes zero and the charges are totally conserved.

### 2.3 Controlled Generation of Multiple Spikes in M‐TENGs

To investigate the effects of contact pair materials on triboelectric signals, we prepared two types of 3‐TENGs with different layer sequences (PDMS–FBP–PDMS and FBP–PDMS–FBP) and compared their spike patterns using *J*_sc_ (Figure S9, Supporting Information). We found that 3‐TENG with the PDMS–FBP–PDMS structure showed indistinct spikes during contact (Figure S9a, Supporting Information), whereas the 3‐TENG with the FBP–PDMS–FBP structure displayed clear spikes during both contact and separation processes (Figure S9b, Supporting Information). The distinct spikes of the FBP–PDMS–FBP structure can be attributed to the superior charge storage capacity of FBP compared to PDMS. FBP can accumulate more charges through the MWS interfacial polarization effect of the BTO nanoparticles, leading to enhanced triboelectric performance (Figure S2c, Supporting Information). This implies that FBP can maintain a high tribo‐negative property throughout the entire cycle of the contact‐separation process. Furthermore, the difference in electron affinity between Al and FBP is greater than that between Al and PDMS (Figure S2e, Supporting Information), which makes FBP a more suitable counter material against Al. Based on these findings, we used FBP as the first contact material in this study to generate distinct multiple spikes.

**Figure 3:**
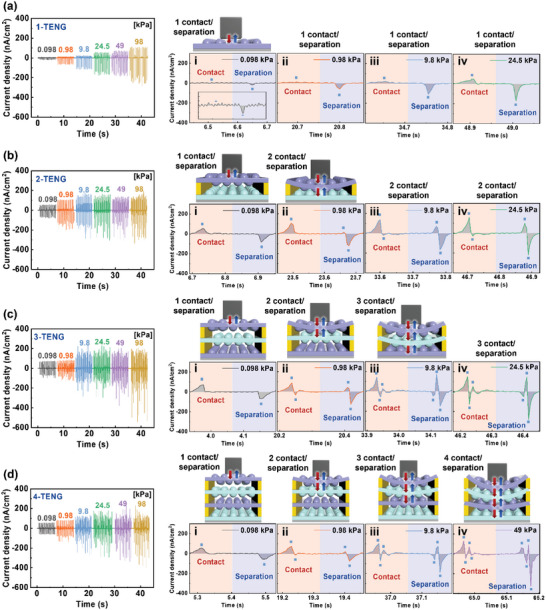
Generation of multiple spikes in M‐TENGs by a single touch. Current densities of a) 1‐TENG, b) 2‐TENG, c) 3‐TENG, and d) 4‐TENG devices under various applied pressures (i) 0.098, ii) 0.98, iii) 9.8, and iv) 24.5 or 49 kPa). Blue dots indicate the distinct peaks of multiple spikes. The schematic insets show that as pressure increases, the friction layers sequentially make contact from top to bottom and separate from bottom to top, inducing distinct spikes in each stage of the contact and separation processes.

M‐TENGs are capable of generating distinct multiple spikes, and the number of spikes can be controlled by varying the applied pressures. **Figure** **3** shows the *J*_sc_ of M‐TENGs with different numbers of layers under various applied pressures at a frequency of 2 Hz. As with conventional TENGs, 1‐TENG generates only two spikes (marked by blue dots) through the triboelectric interaction between FBP and Al (Figure 3a i–iv). However, unlike 1‐TENG, 2‐TENG exhibits a varying number of spikes depending on the applied pressure. At 0.098 kPa, when Al makes contact with the top FBP layer of 2‐TENG, two spikes appear during the one contact/separation process (Figure 3b‐i). As the applied pressure increases from 0.98 to 9.8 kPa, four spikes start to appear when FBP approaches the PDMS film during the two contact/separation processes (Figure 3b‐ii,iii). Consequentially, four distinct spikes are generated at 24.5 kPa (Figure 3b‐iv). Similarly, the number of spikes in 3‐TENG gradually increases with increasing applied pressures (Figure 3c). However, unlike the other cases, the *J*_sc_ of 3‐TENG at different applied pressures exhibits significant variation. At 0.098 kPa, when Al contacts the top PDMS layer, two spikes appear during the one contact/separation process (Figure 3c‐i). Four spikes are generated when FBP contacts PDMS during the two contact/separation processes at 0.98 kPa (Figure 3c‐ii). Finally, during the three contact/separation processes under 9.8 and 24.5 kPa, six spikes appear during the contact/separation process (Figure 3c‐iii,iv). The behavior of 4‐TENG is shown in Figure 3d). Initially, at 0.098 kPa when Al contacts the top FBP layer, two spikes are generated (Figure 3d‐i). Then, four spikes appear when Al induces the contact between the first FBP and second PDMS layers under 0.98 kPa (Figure 3d‐ii). As the pressure further increases to 9.8 kPa, six spikes emerge during the three contact/separation processes (Figure 3d‐iii). Finally, eight spikes are generated in the four contact/separation processes under 49 kPa (Figure 3d‐iv). Additional digital images of the step‐by‐step contact process under various applied pressures can be found in Figure S10, Supporting Information. The *J*_sc_ and *V*_oc_ of M‐TENGs under other magnitudes of pressure are shown in Figures S11 and S12, Supporting Information, respectively.

### 2.4 Energy Harvesting and Sensing Performances of M‐TENGs

Increasing the number of friction layers not only generates multiple spikes but also enhances the overall triboelectric output performance. From the voltage‐charge‐displacement (*V–Q–x*) relationship and the total energy stored in the capacitors of the equivalent circuit,^[^ ^48^ ^]^ we can anticipate the change in the output performance of M‐TENG as the number of layers increases. For better understanding, theoretical models of M‐TENGs are depicted in Figure S13, Supporting Information and discussed in Note S1, Supporting Information. The equation for the total energy stored in a capacitor (*W*) is as follows.

(1) $$W=\frac{1}{2}CV_{oc}^{2}=\frac{Q^{2}}{2C}\left(∵V=\frac{Q}{C}\;\;and\;\;V_{oc}=0\right)$$

where *Q* is the transferred charge and *C* is the capacitance. Then the total energy stored in M‐TENG (*W*_*M* − *TENG*_) can be expressed as follows.

(2) $$W_{1-TENG}=\frac{Q^{2}}{2\frac{\varepsilon_{0}S}{2d_{0}+x_{air}\left(t\right)}}=\frac{Q^{2}\left(2d_{0}+x_{air}\left(t\right)\right)}{2\varepsilon_{0}S}$$

(3) $$W_{2-TENG}=\frac{Q^{2}}{2\frac{\varepsilon_{0}S}{3d_{0}+d_{spacer}+x_{air}\left(t\right)}}=\frac{Q^{2}\left(3d_{0}+d_{spacer}+x_{air}\left(t\right)\right)}{2\varepsilon_{0}S}$$

(4) $$W_{3-TENG}=\frac{Q^{2}}{2\frac{\varepsilon_{0}S}{4d_{0}+2d_{spacer}+x_{air}\left(t\right)}}=\frac{Q^{2}\left(4d_{0}+2d_{spacer}+x_{air}\left(t\right)\right)}{2\varepsilon_{0}S}$$

(5) $$W_{4-TENG}=\frac{Q^{2}}{2\frac{\varepsilon_{0}S}{5d_{0}+3d_{spacer}+x_{air}\left(t\right)}}=\frac{Q^{2}\left(5d_{0}+3d_{spacer}+x_{air}\left(t\right)\right)}{2\varepsilon_{0}S}$$

where *ε_0_* is the vacuum permittivity, *S* is the surface area, *d*_0_ is the effective thickness constant defined as $\sum_{i}^{n}\frac{d_{i}}{\varepsilon_{i}}$, *d_spacer_* is the spacer thickness, and *x_air_* (*t*) is the time‐dependent distance between Al and FBP layer. According to Equations ((2), (3), (4), (5)), the output performance of M‐TENG should increase proportionally to the number of layers‐related terms (i.e., *d*_0_ and *d*_spacer_).

**Figure 4:**
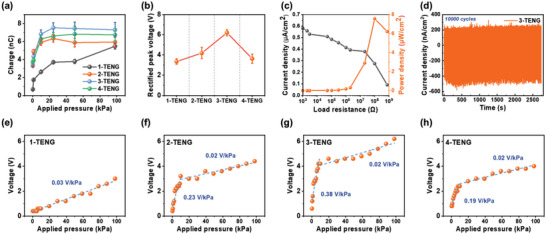
Triboelectric energy harvesting and sensing performances of M‐TENGs. a) Output charge at different applied pressures (0.098–98 kPa) and b) rectified peak voltages of M‐TENGs at 98 kPa. Error bars denote SD from 50 different trials. c) *J*_sc_ and power density of 3‐TENG under different load resistances. d) Cyclic stability of 3‐TENG under 10 000 cycles at 98 kPa. e–h) Pressure‐dependent output voltages and pressure sensitivities of e) 1‐TENG, f) 2‐TENG, g) 3‐TENG, and h) 4‐TENG devices. The pressure sensitivities were obtained through a linear fitting.

**Figure** **4** summarizes several performance indicators of M‐TENGs obtained experimentally for a detailed comparison. One of the significant performance indicators is the output charge (Figure 4a), which was calculated using the following equation.

(6) $$Q=\int I_{sc}dt$$

where *Q* is the output charge, *I_sc_* is the short‐circuit current, and *t* is the measurement time. The output charge serves as an intuitive metric for evaluating the triboelectric performance and compensating for the irregular numerical values of multiple spikes. Initially, at 0.098 kPa, when Al makes contact solely with the uppermost FBP layer in all M‐TENG configurations, the output charges of 2‐TENG, 3‐TENG, and 4‐TENG are similar to one another but significantly higher than that of 1‐TENG. This can be attributed to the difference in the structure of the uppermost FBP layer between 1‐TENG and the other configurations; 1‐TENG has a single‐sided micropattern while the others have bifacial micropattern structures. The bifacial micropattern leads to an increase in the thickness of the friction layer, which subsequently results in a higher output charge, as explained in the aforementioned theoretical model. Moreover, having a larger number of friction layers helps maintain an appropriate distance between electrodes, which prevents charge recombination that could otherwise reduce the triboelectric performance. At pressure above 9.8 kPa, where the 3^rd^ set of contacts is established for 3‐TENG, the output charge of 3‐TENG surpasses that of 2‐TENG. This result is due to the difference in the integration area in the *J*_sc_ data between a single peak and multiple peaks, where enlarged multiple peaks result in an enhanced output charge because of the larger overall integration area. However, the fabrication of 4‐TENG did not prove to be advantageous, as neithe*r J*_sc_ nor the output charge exhibited a significant increase. Contrary to our initial expectation based on the theoretical models described earlier, the output performance of 4‐TENG was found to be inferior to that of 3‐TENG despite having an additional layer. As a result, the highest output charge of 7.52 nC was observed in 3‐TENG, whereas that of 4‐TENG was 6.73 nC at 24.5 kPa. The diminished output performance of 4‐TENG compared to 3‐TENG, can be elucidated by the effective relative permittivity.

(7) $$\varepsilon_{eff}=\left(1-a\right)\varepsilon_{friction}+a\varepsilon_{air}$$

where *ε_eff_* is the effective relative permittivity, *a* is the fraction of air, *ε_friction_* is the relative permittivity of the friction layer, and *ε_air_* is the relative permittivity of air. As shown in Figure S14, Supporting Information, the relative permittivities of PDMS and FBP at 1 kHz are 3.77 and 4.26, respectively, which are higher than that of air (*ε_air_* ∼ 1). In the multi‐layered structure of M‐TENGs, the addition of bifacial micropatterned friction layers results in an increase in the air fraction owing to the air gap between the two micropatterned layers. Therefore, once M‐TENG reaches a certain number of layers, the fraction of air surpasses the fraction of friction layers because the thickness of the air gap (550 µm) is greater than that of the friction layer (≈200 µm), leading to a low effective relative permittivity. More importantly, the triboelectric charges accumulated on each layer may dissipate into the air, especially considering the presence of water molecules in air‐dominant environments.^[^ ^49^, ^50^ ^]^ In this study, the output performance began to decline when the number of layers reached four, with 3‐TENG demonstrating the best output performance. Figure 4b shows the peak voltages of M‐TENGs at 98 kPa, which were rectified by a full‐wave bridge rectifier (Figure S15, Supporting Information). Among the M‐TENGs, 3‐TENG produced the highest rectified peak voltage of 6.2 V, which is sufficiently high for powering electrical devices. On the other hand, the rectified peak voltage of 4‐TENG declined to 3.63 V. This phenomenon arises from surface charge density (σ) which critically affects the *V_OC_*.^[^ ^51^ ^]^

(8) $$V_{OC}=\frac{\sigma x\left(t\right)}{\varepsilon_{0}}$$

where *x*(*t*), and ε_0_ indicate the separation distance, and vacuum permittivity, respectively. On the other hand, in a parallel‐plate capacitor model, the surface charge density can be related to the capacitance of the dielectric layer as follows.

(9) $$\sigma =\frac{CV_{OC}}{S}=\frac{\varepsilon_{0}\varepsilon_{r}}{d}V_{oc}\left(∵C=\frac{S\varepsilon_{r}\varepsilon_{0}}{d}\right)$$

where *C*, *S*, ε_*r*_, and *d* indicate the capacitance, contact area, relative permittivity, and thickness of dielectric layer, respectively. From this relationship, an increase in the ε_*r*_ can enhance the capacitance of the dielectric layer, thereby resulting in an increase in σ. Therefore, the increase in the relative permittivity can enhance the triboelectric voltage.

The output charges and rectified peak voltages of 3‐ and 4‐TENG showed a discrepancy in trend, which is attributed to their respective calculation methods. The rectified peak voltage in Figure 4b represents the highest individual peak among multiple voltage peaks within a contact‐separation cycle. On the other hand, the output charge, derived from Equation (6), takes into account the cumulative effect. It is worth noting that M‐TENGs generate multiple spikes that correspond to the layer number. Since the charge is a cumulative result of the multiple spikes, the total charge of 3‐TENG and 4‐TENG accumulated in one contact‐separation cycle is not as significant as the observed output voltage, even though the highest individual current peak for 3‐TENG is greater than that of 4‐TENG. We adopted these two distinct methods because total charge is important for evaluating energy harvesting performance, whereas individual rectified voltage peak is crucial to assess the synaptic transistor's gate‐source effectiveness.

We further evaluated the energy harvesting performance of 3‐TENG by measuring the output current density and power density (*P*) of 3‐TENG at 98 kPa, with various load resistances (*R_L_*) ranging from 0 to 750 MΩ (Figure 4c). The power density was calculated using the following equation.

(10) $$P=\frac{I^{2}R_{L}}{A}$$

where *A* is the area under pressure. The output power density initially increased, reaching a peak value of 7.56 µW cm^−2^ at 100 MΩ, and then declined as the load resistance continued to increase. The obtained output power density was sufficient to light up 5 blue light‐emitting diodes (LEDs) (Figure S16, Supporting Information). To evaluate the mechanical stability of 3‐TENG for long‐term operation, we subjected 3‐TENG to pressure cyclic tests at 98 kPa (Figure 4d). Throughout 10 000 repeated press‐release cycles, the *J*_sc_ of 3‐TENG remained nearly constant, indicating the stability of 3‐TENG in response to mechanical deformation.

The output voltage produced by M‐TENG increases in proportion to the applied pressure, enabling it to discern the magnitude of the pressure applied. Figure 4e–h shows the pressure sensitivities of M‐TENGs, obtained from the linear fit of the variation in the output voltage with applied pressures ranging from 0.098 to 98 kPa. Pressure sensitivity can be segmented into two regions: low‐pressure (0.098–9.8 kPa) and high‐pressure range (9.8–98 kPa). The magnified pressure‐dependent output voltages and pressure sensitivities of M‐TENGs at the low‐pressure range are shown in Figure S17, Supporting Information. In the low‐pressure range, 1‐TENG did not exhibit any notable pressure sensitivity. However, as the pressure increased, it began to show changes in the output voltage, eventually exhibiting a linear pressure sensitivity in the high‐pressure range with a rate of 0.03 V kPa^−1^. On the other hand, 2‐TENG, 3‐TENG, and 4‐TENG displayed variable pressure sensitivities across the low‐ and high‐pressure ranges. In the low‐pressure range, the pressure sensitivities of 2‐TENG, 3‐TENG, and 4‐TENG were 0.23, 0.38, and 0.19 V kPa^−1^, respectively. However, in the high‐pressure range, the increase in pressure led to relatively low sensitivities as 0.02, 0.01, and 0.02 V kPa^−1^ for 2‐TENG, 3‐TENG, and 4‐TENG, respectively, values that were comparable to that of 1‐TENG. A reasonable explanation for the decrease in the pressure sensitivities in the high‐pressure range can be attributed to the variation in the contact distance and area between the friction layers. In the low‐pressure range, the contact distance between the friction layers continuously decreases as the pressure increases, leading to distinct changes in the output voltage with high‐pressure sensitivities. Notably, 3‐TENG exhibited the highest pressure sensitivity in the low‐pressure range among M‐TENGs, which aligns with the trends observed in the output charge and voltage. However, in the high‐pressure range, where contacts between the friction layers are already established, the contact area changes minimally with increasing pressure, resulting in low‐pressure sensitivities.

### 2.5 M‐TENGs with Multiple Spikes for an Artificial Synaptic Device

The human sensory system processes external mechanical stimuli by using bioelectrochemical signals that neurotransmitters transmit along synapses to the brain. Mechanoreceptors detect these stimuli, which are then translated into sensory information and stored in short‐term memory. Repetitive exposure to stimuli strengthens the connection between pre‐synaptic and post‐synaptic neurons, known as synaptic weight, facilitating the consolidation of memory and the conversion of short‐term memory into long‐term memory.^[^ ^52^ ^]^ This synaptic process can be employed in the development of computational processing and low‐power memory devices by emulating the somatosensory mechanism.^[^ ^53^ ^]^ By integrating M‐TENGs, which can generate multiple spikes with a single touch, with OECTs in an artificial synaptic system, a neuromorphic device with low energy consumption can be developed. Optimizing the structural design to enhance the frequency of multiple spikes in M‐TENGs enables efficient stimuli superposition, which simulates memory training in the biological nervous system.

OECTs have been widely used as artificial synaptic devices because of their low operation voltage resulting from the large capacitance generated by the electric double‐layer (EDL) formation and their non‐volatile memory characteristics originating from the electrochemical doping of ions into the channel.^[^ ^54^, ^55^ ^]^ In particular, ion migration in OECTs is analogous to the transfer of neurotransmitters in biological systems, making OECTs a promising technology for developing artificial neural systems. In this study, we fabricated an OECT using a composite of organic polymer poly(vinylidene fluoride‐trifluoroethylene) (P(VDF‐TrFE)) and an ionic liquid 1‐ethyl‐3‐methylimidazolium bis(trifluoromethylsulfonyl)imide ([EMIM][TFSI]) as the electrolyte layer, poly(3‐hexylthiophene‐2,5‐diyl) (P3HT) as the channel, and Pt as the gate electrode. We chose P(VDF‐TrFE) as the main matrix for the solid polymer electrolyte layer because of its compatibility with ionic liquids and its remnant polarization property, which is advantageous for achieving a long retention time.^[^ ^56^, ^57^ ^]^ P3HT, a conjugated polymer with a thiophene backbone and alkyl side chains, allows for the permeability of ions upon bias voltage application, enabling electrochemical doping operation.^[^ ^58^ ^]^

When a negative voltage is applied to the gate electrode (i.e., Pt), positive ions (i.e., [EMIM]^+^) in the solid polymer electrolyte (i.e., P(VDF‐TrFE)/[EMIM][TFSI]) are drawn toward the gate electrode/solid polymer electrolyte interface, while negative ions (i.e., [TFSI]^−^) migrate to the solid polymer electrolyte/channel interface, forming an EDL at each interface. With higher negative gate voltage (*V*_g_), the accumulated negative ions at the solid polymer electrolyte/channel interface are electrochemically doped into the channel (i.e., P3HT). The doped negative ions induce more holes from the source to the channel to maintain the charge neutrality, leading to enhancement of the drain current (*I*_ds_) (i.e., EPSC).^[^ ^59^, ^60^, ^61^ ^]^ When the negative gate bias is removed, the diffused ions cannot immediately move back to the solid polymer electrolyte, resulting in the gradual decay of EPSC and exhibiting STP features. When the presynaptic spikes are repeated, a larger number of ions become deeply trapped in the crystalline region of P3HT and cannot easily diffuse out even when the gate bias is removed, showing LTP behavior.^[^ ^62^ ^]^ The structure of OECT is illustrated in Figure S18a, Supporting Information. The OECT performance was first characterized without the integration of M‐TENG. A large hysteresis (i.e., memory effect) was observed in the transfer curve (*I*_ds_–*V*_g_) of *V*_g_ = ±2 V, with various sweep rates (Figure S18b, Supporting Information) and among different *V*_g_ (±2 V to ±6 V) with a fixed sweep rate of 1680 mV/s (Figure S18c, Supporting Information). This hysteresis represents the non‐volatile characteristics of the OECT, which is essential for emulating biological synapse functions. Furthermore, the transfer characteristics confirm that the output voltages of M‐TENGs (≈3 to 6 V) align with the gate operation voltage of the OECT.

**Figure 5:**
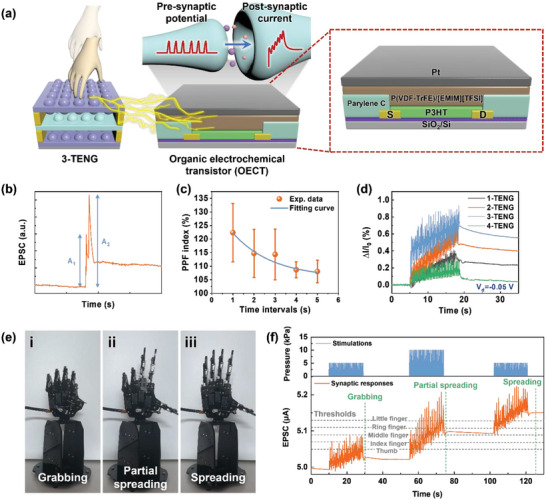
Application of 3‐TENG as an artificial synaptic device and its synaptic characteristics. a) A schematic illustration of an artificial synaptic device consisting of 3‐TENG and an OECT. b) An EPSC demonstrating PPF behavior, induced by two consecutive pressures (98 kPa, *V*_ds_ = −0.05 V). c) An experimentally plotted PPF index and a fitting curve at various time intervals (98 kPa). Error bars denote the standard deviation from 50 different trials. d) EPSCs of 1‐, 2‐, 3‐ and 4‐TENG under 50 stimulations (98 kPa, 2 Hz and *V*_ds_ = −0.05 V). e) Digital photos of a robotic hand in three states: i) grabbing, ii) partial spreading, and iii) fully spreading, as operated by different EPSCs. f) Memory training process of the robot hand under weak (5 kPa) and strong (10 kPa) external stimulations.

To realize an artificial synapse that operates with triboelectric potential gating, 3‐TENG was integrated with the OECT (**Figure** **5**a). Under external stimuli, 3‐TENG, functioning as a mechanoreceptor, produced voltage signals with multiple spikes. With a full‐wave bridge rectifier, the generated voltage was converted to negative pre‐synaptic voltage (i.e., *V*_g_), which was then used to control the gate of the OECT (pre‐synaptic neuron). As a result, the *I*_ds_ of OECT, which represents the EPSC, was modulated in response to the external pressure. The synaptic characteristics of 3‐TENG integrated OECT were examined by simulating paired‐pulse facilitation (PPF) behavior through the application of two consecutive pressures to 3‐TENG (Figure 5b). PPF behavior, known as a form of STP, enhances the synaptic connection during the process of neurotransmission.^[^ ^63^ ^]^ The drain‐source voltage (*V*_ds_) was maintained at −0.05 V. The 3‐TENG integrated OECT shows a typical PPF behavior, where the second EPSC spike (*A_2_*) is larger than the first EPSC spike (*A_1_*). The PPF index, a measure of how effectively the synapse facilitates communication between neurons, is defined as follows.

(11) $$PPF\left(\%\right)=\frac{A_{2}}{A_{1}}\times 100$$

Based on a couple of EPSC spikes, the time‐interval‐dependent PPF index was plotted in Figure 5c). The fitting curve of the PPF index can be expressed by the following equation.

(12) $$y=y_{0}+B_{1}exp\left(\frac{-\Delta t}{\tau_{1}}\right)+B_{2}exp\left(\frac{-\Delta t}{\tau_{2}}\right)$$

where *y_0_* is the resting facilitation magnitude, *B_1_* and *B_2_* are the facilitation constants, *τ_1_* and *τ_2_* are the relaxation times, and *∆t* is the spike interval time, respectively. In this case, *y_0_*, *τ_1_*, and *τ_2_* were 99.39, 1.34 s, and 8.3 s, respectively. The PPF index gradually increased to 122% as the time interval between stimulations was shortened, which was also shown in a fitting curve corresponding to these plots. To assess the effectiveness of using the 3‐TENG in artificial synaptic device applications, the changes in EPSC (*∆I/I_0_*) were compared for different M‐TENG configurations (1‐TENG, 2‐TENG, 3‐TENG, and 4‐TENG) during 50 stimulations at 98 kPa and 2 Hz (Figure 5d). The EPSC increased significantly with an increase in the number of TENG layers until 3‐TENG and then decreased for 4‐TENG, which was due to the low rectified voltage of 4‐TENG (Figure S15, Supporting Information). Unlike the trends in Figure 4e,h, the EPSC change of 1‐TENG was higher than that of 4‐TENG. From the enlarged plot of rectified voltage for 1‐TENG and 4‐TENG (Figure S19, Supporting Information), it can be observed that 4‐TENG exhibits a larger number of peaks. However, each peak interval is narrower, and smaller voltage peaks are also evident. To evaluate the effect of voltage waveform on the drain current of the transistor, we simulated two types of waveforms, which mimic the rectified output voltage of 1‐TENG (Figure S19a, Supporting Information) and 4‐TENG (Figure S19b, Supporting Information). Then, we applied gate voltages using these two waveforms via a function generator and compared the resulting changes in drain current (Figure S19c–e, Supporting Information). The waveform mimicking 1‐TENG induced a more significant change in current compared to the waveform emulating 4‐TENG, which is attributed to the small voltage peaks present within the cycle for 4‐TENG. The EPSC change of 0.9% with 3‐TENG was the highest due to the large gate voltage input with high frequency. Once the stimulations were stopped, the EPSC was maintained at a certain level, indicating a tendency to develop LTP.

We utilized the synaptic behavior of the 3‐TENG integrated OECT to operate a robotic hand and simulate its memory training, also known as associative learning. The robotic hand was activated when the EPSC exceeded a certain threshold set by an Arduino circuit. The detailed circuit configuration is shown in Figure S20, Supporting Information. The operation of a robotic hand could be expressed in three states: grabbing, partial spreading, and fully spreading (Figure 5e). We applied weak (5 kPa) and strong (10 kPa) stimulations at a frequency of 2 Hz, and designated EPSC thresholds corresponding to the thumb, index finger, middle finger, ring finger, and little finger as 5.05, 5.07, 5.09, 5.11, and 5.13 µA, respectively (Figure 5f) and Video S1, Supporting Information). First, when 50 weak stimulations were applied, some of the robotic fingers spread and folded back and forth due to the real‐time spiky EPSC, and eventually, all the robotic fingers remained folded because the EPSC was below the threshold with a low synaptic weight (grabbing state). Then, after applying 50 strong stimulations, the robotic fingers were partially spread as the EPSC approached the intermediate thresholds (partial spreading state). When 50 weak stimulations were applied again, the elevated EPSC from the previous strong stimulations caused the synaptic weight to exceed all thresholds, resulting in the robotic hand fully spreading (fully spreading state). This associative learning experiment simulates the memory training process of the human sensory system. Before the training process, the robotic hand did not respond well to weak stimuli, leading to easy forgetting of sensory information. During the training process, the system converts external stimuli into synaptic responses. After the training process, the establishment of LTP could be accomplished through weak stimuli, due to previously excited synaptic responses by continuous external stimuli. These processes strengthen the synaptic connection.

## 3 Conclusion

In summary, we successfully developed M‐TENGs capable of generating multiple triboelectric spikes under mechanical stimuli. Unlike conventional TENG (referred to as 1‐TENG in this study), M‐TENGs generate distinct multiple spikes corresponding to the number of layers with a single touch. The generation of multiple spikes is achieved through the use of spacers that create an instantaneous contact/separation delay between two friction layers. Additionally, the micropatterns on the surface of each layer result in low adhesive strength during separation, which effectively reduces the damping phenomenon and facilitates the generation of distinct spikes. The number of spikes can be systematically controlled by adjusting the number of friction layers and the pressure applied. It was observed that the 3‐TENG configuration exhibited the highest triboelectric output performance, with an output charge of 7.52 nC, a rectified output voltage of 6.2 V, and an output power of 7.56 µW cm^−2^ at 100 MΩ under 98 kPa. To capitalize on its unique triboelectric output characteristics of generating multiple spikes, the 3‐TENG was integrated with an OECT, creating an energy‐efficient artificial synaptic device. This device emulates the synaptic functions of biological synapses, such as STP and LTP, through mechanical stimuli. Consequently, a robotic hand was successfully operated and synchronized through a memory training process, which was linked to a real‐time data acquisition circuit. This research demonstrates the potential for applications in next‐generation self‐powered electronics, neuro‐inspired artificial electronics, and HMIs.

## 4 Experimental Section

### Fabrication of Triboelectric Layers with Bifacial Micropatterns

The fabrication process of the triboelectric layers is schematically illustrated in Figure S21, Supporting Information. Briefly, a PDMS precursor and a curing agent (Sylgard 184, Dow Corning, US) were mixed in a 10:1 weight ratio, followed by degassing the PDMS solution in a vacuum desiccator to remove any trapped air bubbles. The PDMS solution was then poured onto a micropatterned mold, spread evenly using spin‐coating at 1000 rpm for 30 s, and cured at 80 °C for 3 h. Subsequently, a single‐sided micropatterned PDMS film (height of 5 µm, diameter of 10 µm, and pitch of 12 µm) was obtained by peeling it off the mold, which was used as a one‐layer triboelectric nanogenerator (1‐TENG). For the fabrication of bifacial PDMS micropatterns, the single‐sided micropatterned PDMS film was placed onto the PDMS that has been spin‐coated on another mold but yet cured. After aligning the PDMS layers, the curing and demolding processes were conducted to obtain bifacial PDMS micropatterns as a tribo‐positive friction layer.

To synthesize FBP with bifacial micropatterns as a tribo‐negative friction layer, BTO nanoparticles (average diameter of ≈200 nm, US Research Nanomaterials, Inc., US) were dispersed in ethanol at different concentrations (0, 1, 5, and 10 wt%) through sonication for 30 min. The BTO‐ethanol dispersion was then mixed with the PDMS base using a planetary centrifugal mixer (ARE‐310, THINKY, US) at 2000 rpm for 15 min and degassed at 2200 rpm for 5 min. The BTO/PDMS base solution was stirred overnight at 100 °C to evaporate ethanol solvent and mixed with a curing agent with a weight ratio of 10:1, followed by the aforementioned curing and demolding processes. For the FOTS coating on the bifacial BTO/PDMS micropatterns, the bifacial surfaces were treated with O_2_ plasma using a plasma cleaner (Tergeo, PIE Scientific LLC, US) to form hydrophilic functional groups on the surfaces. Then, the bifacial micropatterns were coated with FOTS using a self‐assembled monolayer (SAM) coater (AVC‐150, SORONA, South Korea).

### Fabrication of M‐TENG

Multi‐layered TENGs were fabricated on a Ni/Cu electrode, which was adhered to a glass substrate. The distance between each friction layer was controlled using 0.55 mm‐thick polyimide tapes as spacers. To fabricate a 1‐TENG, a single‐sided micropatterned FBP film was attached to the Ni/Cu electrode. For the 2‐TENG, a bifacially micropatterned FBP layer was placed on top of a single‐sided micropatterned PDMS layer, with two spacers on each side for the separation of the films. Similarly, 3‐TENG and 4‐TENG were fabricated by sequentially stacking PDMS or FBP layers. After stacking each upper layer, the layers were stretched to a strain of 15% using adhesive tape on each side, to ensure better reversible contact and separation through the tension in the stretched layer.

### Fabrication of OECT

The detailed fabrication process of OECT is depicted in Figure S22, Supporting Information and described in Note S2, Supporting Information. Briefly, for the source/drain electrodes, Cr/Au interdigitated electrodes were e‐beam evaporated on a SiO_2_/Si wafer. To electrically isolate the electrodes, a 1^st^ parylene C layer was coated on top of the Cr/Au electrodes. Then, a 2^nd^ parylene C sacrificial layer was coated with a Micro‐90 (International Products Corporation) anti‐adhesive layer in between the two parylene C layers. A Ti etch hard mask was deposited on the topmost parylene C layer, followed by reactive ion etching of the Ti and parylene C layers to expose the Cr/Au electrodes. Then, P3HT was spin‐coated on the Cr/Au electrodes as a channel, followed by annealing to evaporate solvents. The topmost parylene C was peeled‐off, and a composite of P(VDF‐TrFE) and an ionic liquid ([EMIM][TFSI]) was spin‐coated on P3HT as an electrolyte layer, followed by annealing to evaporate solvents and enhance crystallinity. Finally, an OECT was completed by depositing a Pt gate electrode.

### Characterizations

All electrical output measurements of the TENGs were conducted in contact‐separation mode under different applied pressures using a pushing tester (JIPT, JUNIL TECH, South Korea) with a contact area of 1 × 1 cm^2^. The output performances were optimized by controlling the contact‐separation distance and spacer thickness (Figures S23 and S24, Supporting Information). The optimum contact‐separation distance and spacer thickness were found to be 2 and 0.55 mm, respectively. The *J*_sc_ and EPSC were measured using a sourcemeter (Model 2450, Keithley, US). The *V*_oc_ was recorded using an oscilloscope (DPO 2022B, Tektronix, US). The transfer and output characteristics of the OECT were measured using a semiconductor parameter analyzer (4200‐SCS, Keithley, US). To operate a robotic hand (Hiwonder, China) under the memory training process, all processes were controlled using the Python language. Real‐time data from the sourcemeter were transmitted to a microcontroller unit (Arduino Uno) that was connected to the laptop in series. Morphological studies of the samples were characterized using a FE‐SEM (Quanta200FEG, FEI, US). Chemical compositions and crystallinity of samples were analyzed by XPS (K‐alpha, ThermoFisher, US) and XRD (D8 ADVANCE, Bruker, US), respectively. The adhesive strength was measured using a texture analyzer (TXATM‐Precision, YEONJIN S‐TECH, South Korea).

## Conflict of Interest

The authors declare no conflict of interest.

## Supporting information

Supporting Information

Supplemental Video 1

## Data Availability

The data that support the findings of this study are available from the corresponding author upon reasonable request.
