# Supplementary material for: Multi‐Layered Triboelectric Nanogenerators with Controllable Multiple Spikes for Low‐Power Artificial Synaptic Devices
Source: Adv Sci (Weinh). 2023 Oct 27;10(36):2304598. doi: 10.1002/advs.202304598 (PMC10754122; doi:10.1002/advs.202304598)
Supplement: Supplementary file 1 — Supporting Information [file ADVS-10-2304598-s001.pdf]

ADVANCED SCIENCE
Open Access

Supporting Information

for *Adv. Sci.*, DOI 10.1002/advs.202304598

Multi-Layered Triboelectric Nanogenerators with Controllable Multiple Spikes for Low-Power Artificial Synaptic Devices

*Yong-Jin Park, Yun Goo Ro, Young-Eun Shin, Cheolhong Park, Sangyun Na, Yoojin Chang and Hyunhyub Ko**

Supporting Information

Multi-Layered Triboelectric Nanogenerators with Controllable Multiple Spikes for Low Power Artificial Synaptic Devices

*Yong-Jin Park †, Yun Goo Ro †, Young-Eun Shin, Cheolhong Park, Sangyun Na, Yoojin Chang and Hyunhyub Ko**

School of Energy and Chemical Engineering, Ulsan National Institute of Science and Technology (UNIST), 50, UNIST-gil, Ulsan, 44919, Republic of Korea
***Corresponding Author**
E-mail: hyunhko@unist.ac.kr

†: These authors contributed equally to this work.

Supplementary Note 1

Theoretical model of M-TENGs

Related equations are shown as below.

$$I_{sc} = \frac{dQ}{dt} = S\frac{d\sigma}{dt} \quad (1)$$

$$V_{oc} = \frac{\sigma x(t)}{\varepsilon_0} \quad (2)$$

$$\sigma = \frac{CV_{OC}}{S} = \frac{\varepsilon_0\varepsilon_r}{d}V_{oc}\ (\because C\ =\ \frac{S\varepsilon_r\varepsilon_0}{d}) \quad (3)$$

where Q is the transferred charge, S is the surface area, σ is the surface charge density, *x* is the distance between friction layers, ε_0 is the vacuum permittivity, ε_r is the relative permittivity and d is the thickness of friction layer. In the case of 1-TENG, electric field strength at each region can be expressed as

$$E_{air} = \frac{-\frac{Q}{S}+\sigma(t)}{\varepsilon_0} = -\frac{Q}{S\varepsilon_0} + \frac{\sigma(t)}{\varepsilon_0} \quad (4)$$

$$E_{FBP} = -\frac{Q}{S\varepsilon_0\varepsilon_{FBP}} \quad (5)$$

According to Gauss' theorem,[1] the voltage between the two electrodes with *V–Q–x* relationship can be given by

$$V_{1-TENG} = E_{FBP}d'_{FBP} + E_{air}x_{air}(t) = -\frac{Q}{S\varepsilon_0}\left(\frac{d'_{FBP}}{\varepsilon_{FBP}} + x_{air}(t)\right) + \frac{\sigma(t)x_{air}(t)}{\varepsilon_0} \quad (6)$$

where d' is the thickness of hemispherical micropatterned film (Fig. S13(a)). In the case of 2-TENG, following terms are added into *V–Q–x* relationship of 1-TENG.

$$E_{spacer} = \frac{-\frac{Q}{S}+\sigma_{spacer}(t)}{\varepsilon_0} = -\frac{Q}{S\varepsilon_0} + \frac{\sigma_{spacer}(t)}{\varepsilon_0} \quad (7)$$

$$E_{PDMS} = -\frac{Q}{S\varepsilon_0\varepsilon_{PDMS}} \quad (8)$$

The voltage between the two electrodes with *V–Q–x* relationship can be depicted by

$$V_{2-TENG} = E_{PDMS}d'_{PDMS} + E_{spacer}d_{spacer}(t) + E_{FBP}d_{FBP} + E_{air}x_{air}(t)$$

$$= -\frac{Q}{S\varepsilon_0}\left(\frac{d'_{PDMS}}{\varepsilon_{PDMS}} + \frac{d_{FBP}}{\varepsilon_{FBP}} + d_{spacer}(t) + x_{air}(t)\right) + \frac{\sigma_{spacer}(t)d_{spacer}(t)}{\varepsilon_0} + \frac{\sigma(t)x_{air}(t)}{\varepsilon_0} \quad (9)$$

In a similar way, the voltages of 3-TENG and 4-TENG are calculated by

$$V_{3-TENG} = E_{FBP}d'_{FBP} + 2E_{spacer}d_{spacer}(t) + E_{PDMS}d_{PDMS} + E_{FBP}d_{FBP} + E_{air}x_{air}(t) =$$

$$-\frac{Q}{S\varepsilon_0}\left(\frac{d'_{FBP}}{\varepsilon_{FBP}} + \frac{d_{PDMS}}{\varepsilon_{PDMS}} + \frac{d_{FBP}}{\varepsilon_{FBP}} + 2d_{spacer}(t) + x_{air}(t)\right) + 2\frac{\sigma_{spacer}(t)d_{spacer}(t)}{\varepsilon_0} + \frac{\sigma(t)x_{air}(t)}{\varepsilon_0} \quad (10)$$

$$V_{4-TENG} = E_{PDMS}d'_{PDMS} + 2E_{FBP}d_{FBP} + 3E_{spacer}d_{spacer}(t) + E_{PDMS}d_{PDMS} + E_{air}x_{air}(t)$$

$$= -\frac{Q}{S\varepsilon_0}\left(\frac{d'_{PDMS}}{\varepsilon_{PDMS}} + 2\frac{d_{FBP}}{\varepsilon_{FBP}} + \frac{d_{PDMS}}{\varepsilon_{PDMS}} + 3d_{spacer}(t) + x_{air}(t)\right) + 3\frac{\sigma_{spacer}(t)d_{spacer}(t)}{\varepsilon_0} +$$

$$\frac{\sigma(t)x_{air}(t)}{\varepsilon_0} \quad (11)$$

For 1-TENG,

$$Q_{sc} = \frac{S\sigma(t)x(t)}{\frac{d'_{FBP}}{\varepsilon_{FBP}}+x(t)} \; (\because V = 0) \quad (12)$$

whereas in the open-circuit case,

$$V_{oc} = \frac{\sigma(t)x_{air}(t)}{\varepsilon_0} \ (\because Q = 0) \tag{13}$$

In terms of capacitors in an equivalent circuit of 1-TENG shown in Fig. S13(b), capacitance of each capacitor is given by

$$C_{air} = C_1 = \frac{Q_{sc}}{V_{oc}} = \frac{\varepsilon_0 S}{\frac{d'_{FBP}}{\varepsilon_{FBP}} + x_{air}(t)} \tag{14}$$

$$C_{FBP} = C_2 = \frac{\varepsilon_0 \varepsilon_{FBP} S}{d'_{FBP}} \tag{15}$$

To simplify the equations, effective thickness constant (d_0) is defined by $\sum_i^n \frac{d_i}{\varepsilon_i}$.

Using equations (14) and (15), we can obtain total capacitance in series as below.

$$C_{total,1-TENG} = \frac{1}{\frac{1}{C_1}+\frac{1}{C_2}} = \frac{1}{\frac{d_0+x_{air}(t)}{\varepsilon_0 S}+\frac{d_0}{\varepsilon_0 S}} = \frac{\varepsilon_0 S}{2d_0+x_{air}(t)} \tag{16}$$

Similarly, the capacitance of PDMS and total capacitance of 2-TENG, 3-TENG and 4-TENG are given by (Fig. S13(c)-(h))

$$C_{PDMS} = \frac{\varepsilon_0 \varepsilon_{PDMS} S}{d_{PDMS}} = \frac{\varepsilon_0 S}{d_0} \tag{17}$$

$$C_{spacer} = \frac{\varepsilon_0 \varepsilon_{spacer} S}{d_{spacer}} = \frac{\varepsilon_0 S}{d_{spacer}} \ (\because \varepsilon_{spacer} = 1)$$

$$C_{total,2-TENG} = \frac{1}{\frac{1}{C_1}+\frac{1}{C_2}+\frac{1}{C_3}+\frac{1}{C_4}} = \frac{1}{\frac{d_0+x_{air}(t)+d_{spacer}+2d_0}{\varepsilon_0 S}} = \frac{\varepsilon_0 S}{3d_0+d_{spacer}+x_{air}(t)} \tag{18}$$

$$C_{total,3-TENG} = \frac{1}{\frac{1}{C_1}+\frac{1}{C_2}+\frac{1}{C_3}+\frac{1}{C_4}+\frac{1}{C_5}+\frac{1}{C_6}} = \frac{1}{\frac{d_0+x_{air}(t)+2d_{spacer}+3d_0}{\varepsilon_0 S}} = \frac{\varepsilon_0 S}{4d_0+2d_{spacer}+x_{air}(t)} \tag{19}$$

$$C_{total,4-TENG} = \frac{1}{\frac{1}{C_1}+\frac{1}{C_2}+\frac{1}{C_3}+\frac{1}{C_4}+\frac{1}{C_5}+\frac{1}{C_6}+\frac{1}{C_7}+\frac{1}{C_8}} = \frac{1}{\frac{d_0+x_{air}(t)+3d_{spacer}+4d_0}{\varepsilon_0 S}} = \frac{\varepsilon_0 S}{5d_0+3d_{spacer}+x_{air}(t)} \tag{20}$$

Supplementary Note 2

OECT fabrication: Source and drain (S/D) interdigitated electrodes with 50 μm spaced 10 fingers were patterned by photolithography on a piranha-cleaned Si wafer with thermally grown SiO_2 (100 nm). Cr/Au (5/50 nm) were deposited by e-beam evaporation as S/D electrodes followed by lift-off process in acetone. 2 layers of 5 μm-thick parylene C layers were coated, the first parylene C as a electrical isolation layer, the second parylene C layer as a sacrificial layer. In between two parylene C layers, Micro-90 (International Products Corporation) solution diluted with deionized water (0.04 vol%) was coated. The Micro-90 solution served as an anti-adhesion layer to facilitate separation of the sacrificial parylene C layer. After O_2 plasma treatment of the parylene C surface, a Ti (100 nm) layer was deposited by e-beam evaporation as an etch mask to etch parylene C layers. Photolithography was performed to define active region of the channel with width of 1000 μm. A photoresist (AZ nLOF 2035, MicroChemicals) was served as an etch mask to etch the Ti layer. The Ti and parylene C layers were etched by reactive ion etching to expose the channel region and the S/D electrodes, followed by solvent cleaning using acetone, isopropanol, deionized water to remove any parylene C or Micro-90 residues on the active region. Poly(3-hexylthiophene-2,5-diyl) (P3HT, regioregular, average M_w=85,000–100,000, Sigma-Aldrich) was dissolved in 1,2-dichlorobenzene (5 mg/ml, Sigma-Aldrich) and stirred for 2 h at 60 °C. The P3HT solution was spin-coated on the S/D electrodes at 2000 rpm, 40 s, followed by 70 °C, 5 min annealing to remove residual solvents. After spin-coating of P3HT, the topmost parylene C layer was peeled-off to leave the P3HT only on the active region. For an electrolyte layer, poly(vinylidenefluoride-trifluoroethylene) (P(VDF-TrFE, Piezotech) powder was dissolved in N,N-dimethylformamide (DMF, Sigma-Aldrich) (10 wt%) and stirred for 2 h at 60 °C. Then 5 wt% of ionic liquid (IL) 1-ethyl-3-methylimidazolium bis(trifluoromethylsulfonyl)imide ([EMIM][TFSI], Sigma-Aldrich) was added to the P(VDF-TrFE)/DMF solution and stirred for 2 h at 60 °C. The P(VDF-TrFE)/IL/DMF solution was spin-coated on top of the P3HT channel at 1000 rpm, 30 s, followed by 60 °C, 30 min and 140 °C, 2 h annealing to remove remaining DMF and enhance the crystallinity of P(VDF-TrFE). Finally, Pt gate electrode was deposited on top of P(VDF-TrFE)/IL by sputtering.

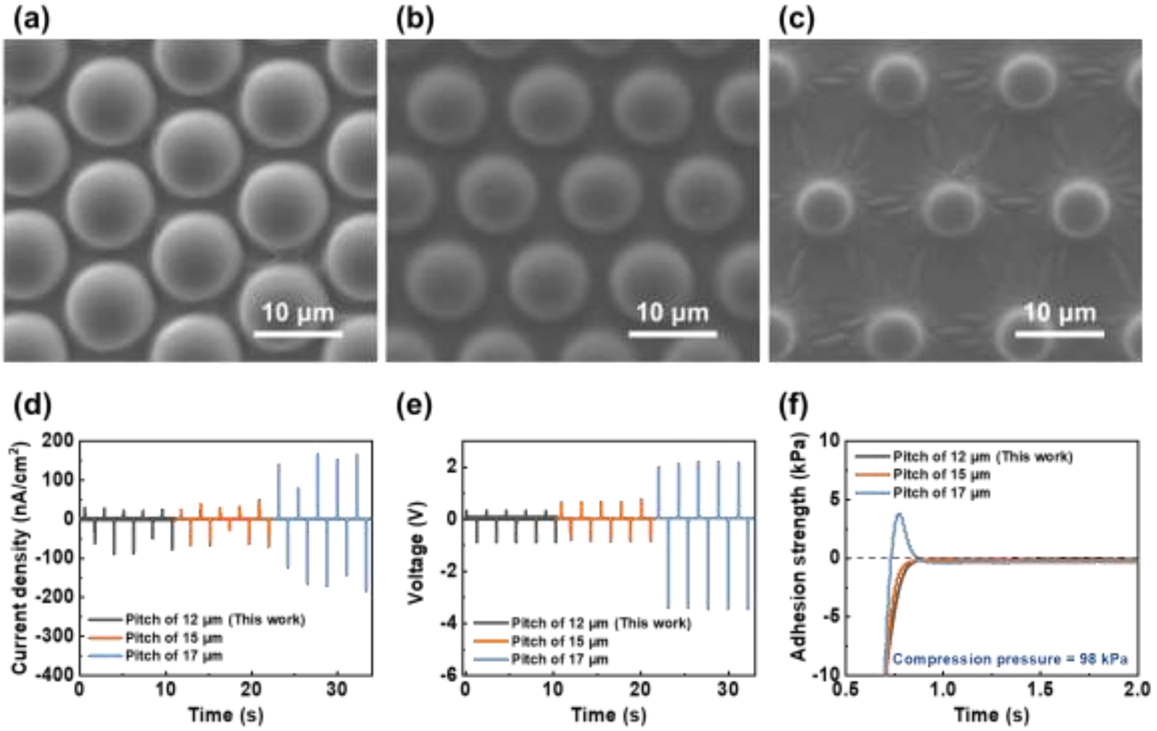

Figure S1. FE-SEM images of the micropatterned PDMS with the different pitches of (a) 12, (b) 15 and (c) 17 µm. Optimization of (d) J_{sc} and (e) V_{oc} of the micropatterned PDMS under an external pressure of 98 kPa. (f) Adhesion strengths of the micropatterned PDMS with the different pitches of 12, 15 and 17 µm against Al at a compression pressure of 98 kPa.

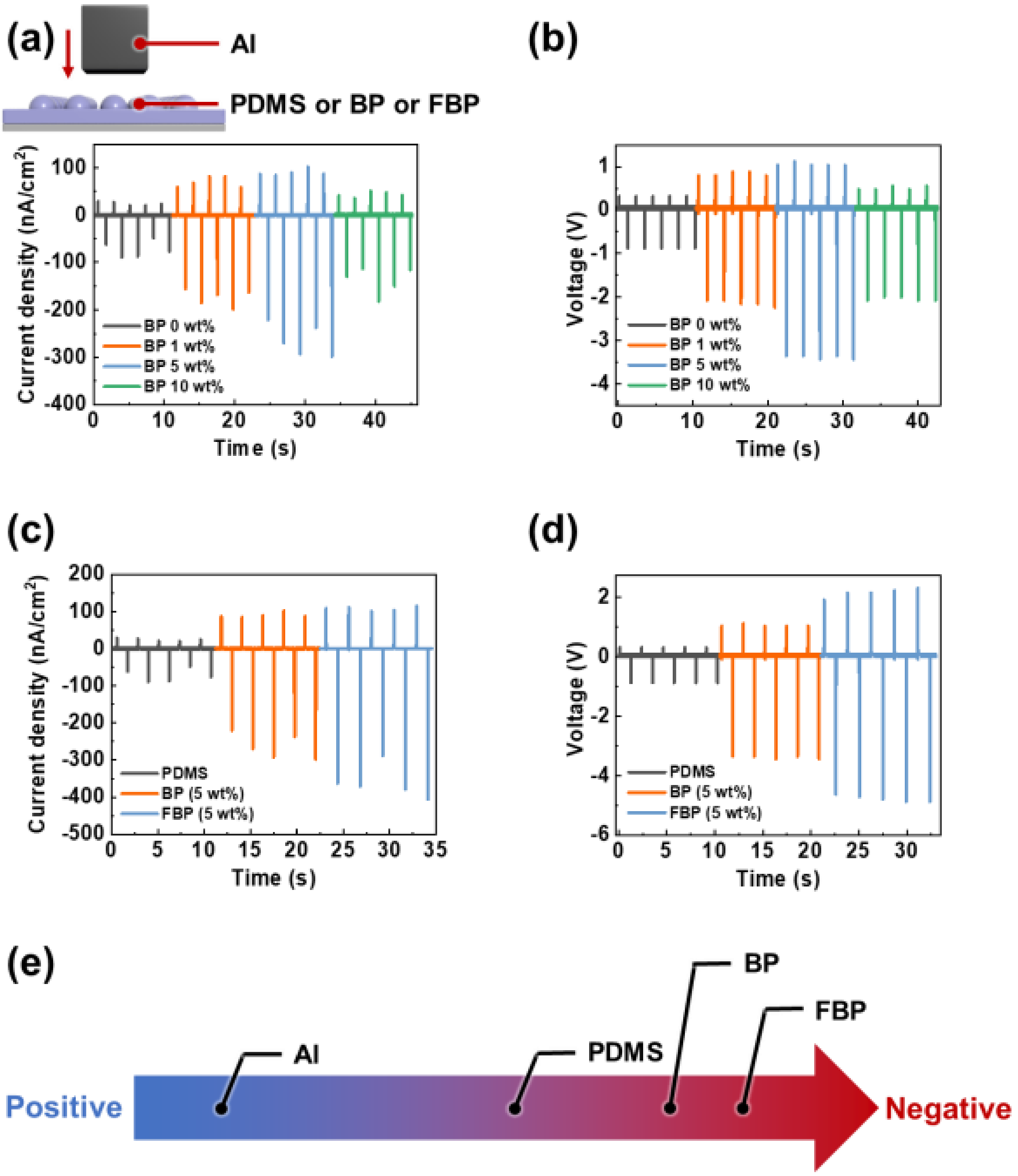

Figure S2. Optimization of triboelectric materials using Al under an external pressure of 98 kPa. (a) J_{sc} and (b) V_{oc} of the BP film with different BTO concentrations. (c) J_{sc} and (d) V_{oc} of PDMS, BP and FBP films. (e) Triboelectric series of triboelectric materials used in this work. Al is located in the tribo-positive region, whereas PDMS, BP and FBP are located in the tribo-negative region.

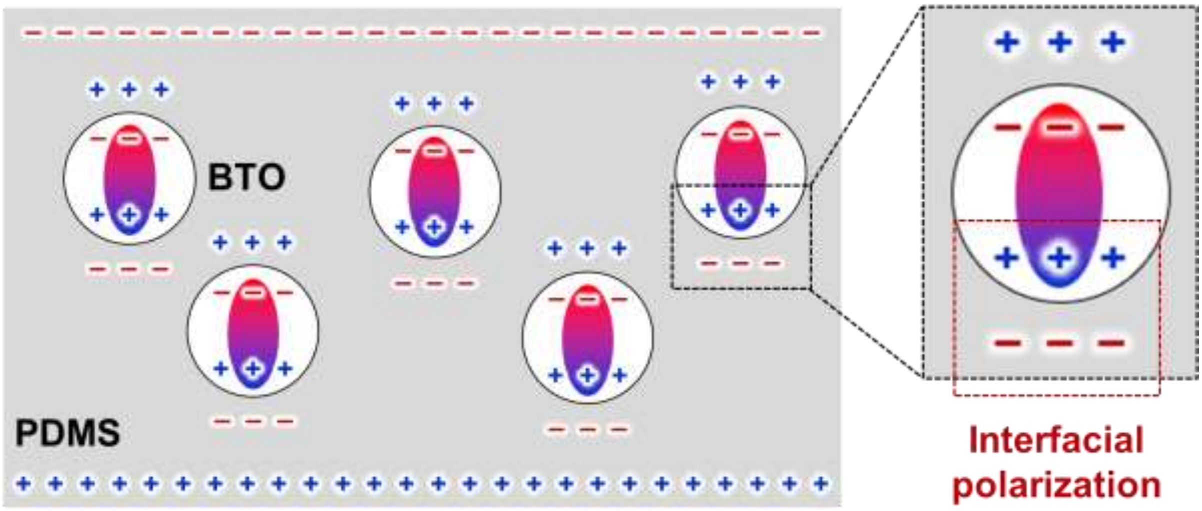

Figure S3. A schematic illustration of the MWS interfacial polarization effect of BTO NPs in PDMS matrix. Additional positive and negative charges are induced at the interface between BTO NP and PDMS matrix due to the aligned dipoles of the BTO NPs. Hence, the charge storage capability and the surface charge density of BP film are enhanced.

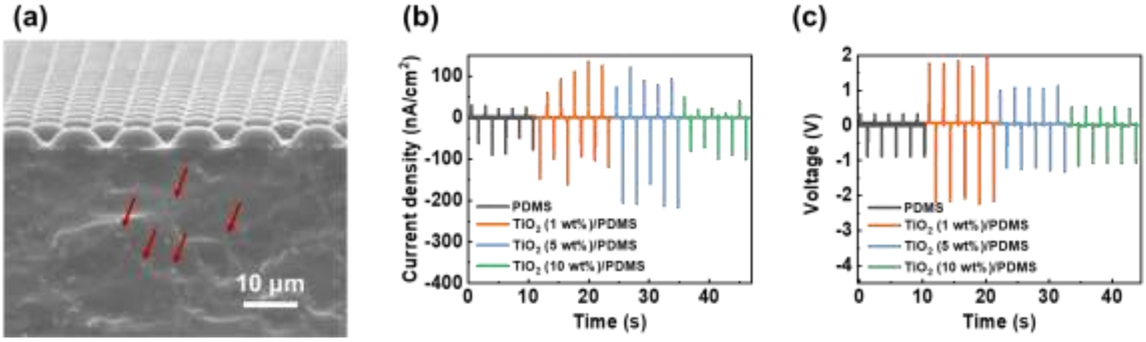

Figure S4. (a) Cross-sectional FE-SEM image of a TiO_2/PDMS composite film with 5 wt% TiO_2 nanoparticles. The red arrows indicate TiO_2 nanoparticles embedded in the PDMS matrix. (b) J_{sc} and (c) V_{oc} of TiO_2/PDMS composite films with different TiO_2 concentrations of 0, 1, 5 and 10 wt%, measured against Al under an external pressure of 98 kPa.

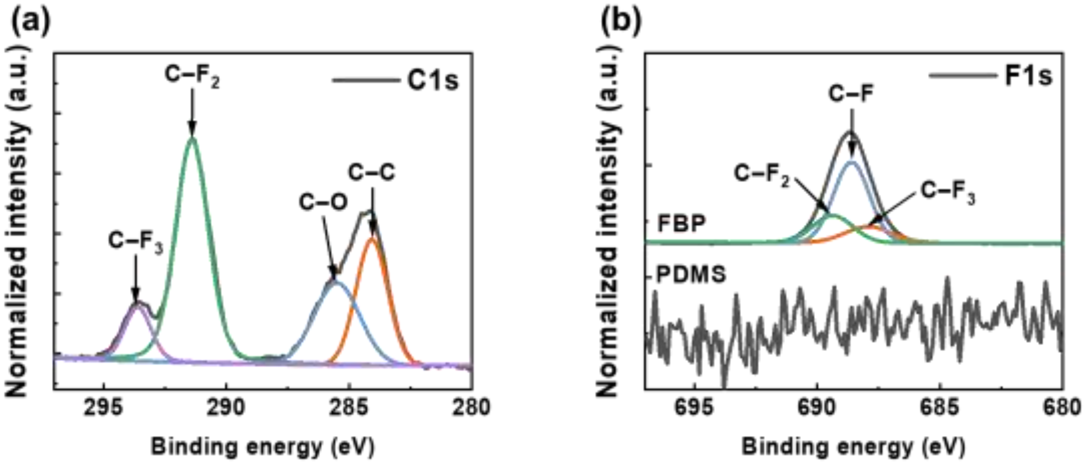

Figure S5. XPS analysis of FBP in the (a) C 1s region and (b) F 1s region. No peaks corresponding to PDMS can be found in F 1s region.

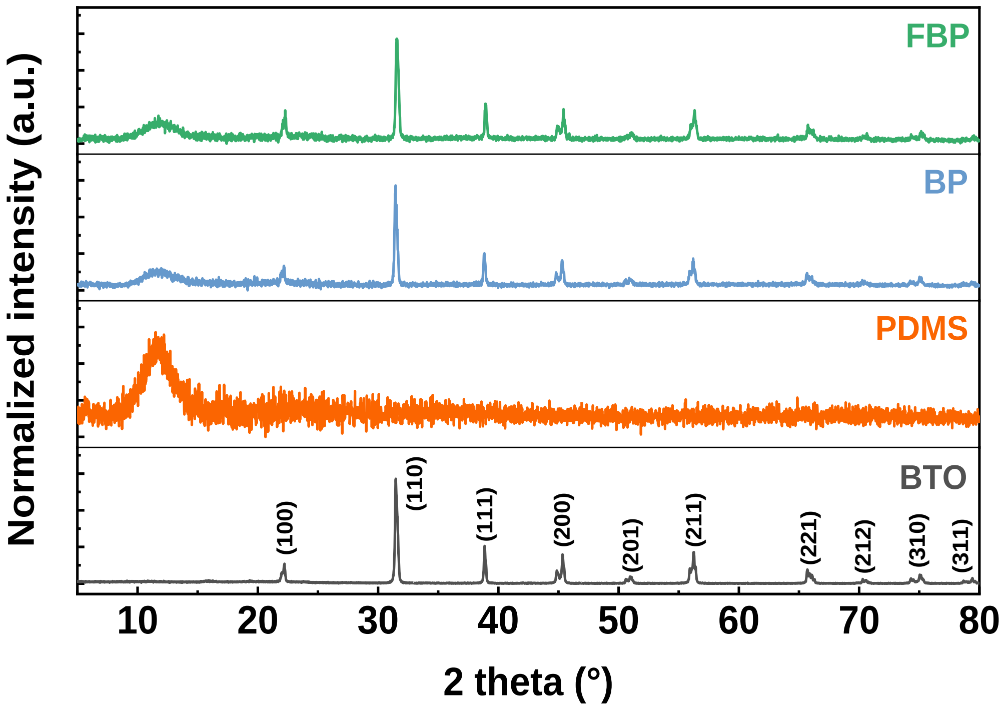

Figure S6. XRD patterns of BTO nanoparticles, PDMS, BP and FBP ranging from 5 to 80°. According to XRD patterns of BP and FBP, the crystalline BTO NPs are evenly embedded within the amorphous PDMS matrix without any deformation of the chemical structure.

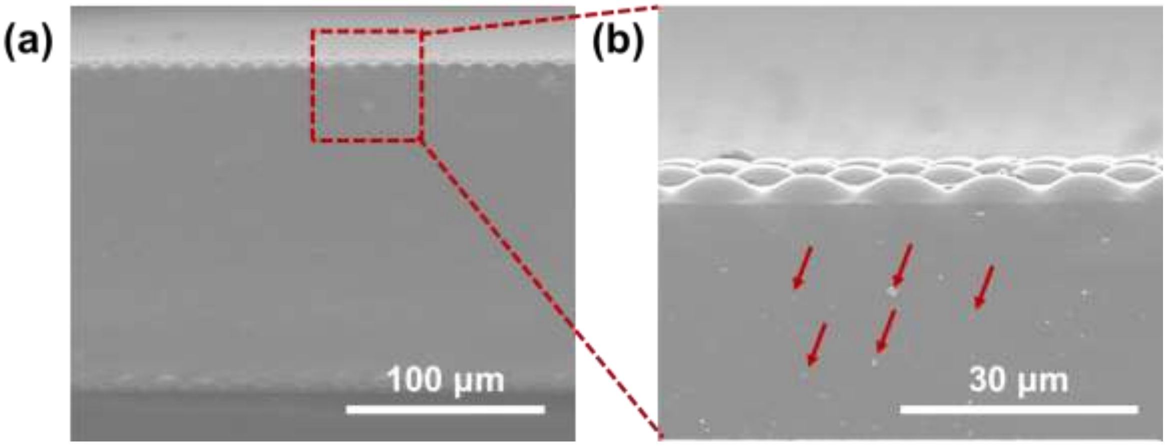

Figure S7. (a) Cross-sectional FE-SEM image and (b) high-magnification image of the micropatterned FBP. The red arrows indicate BTO nanoparticles embedded in the PDMS matrix. The BTO nanoparticles are homogeneously distributed without aggregation.

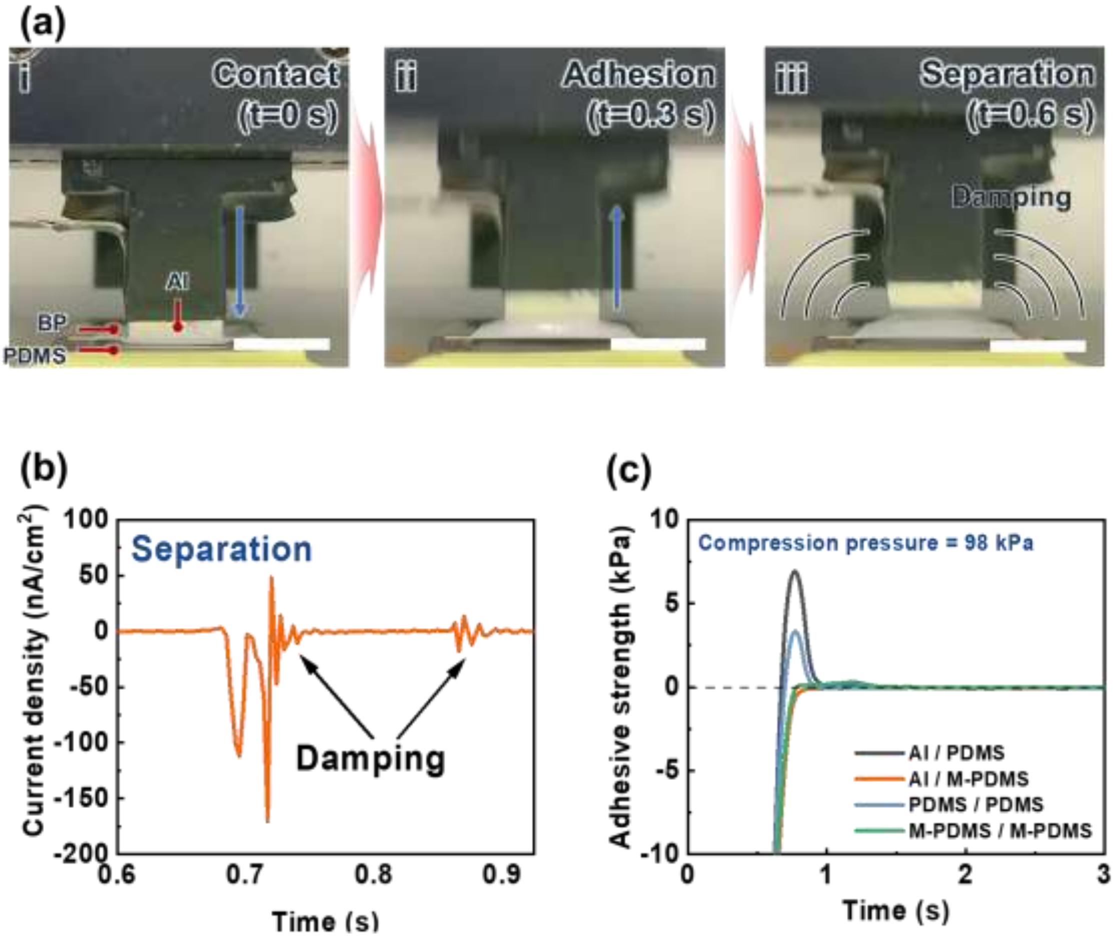

Figure S8. Adhesion test of planar and microdome-patterned films using a pushing tester and a texture analyzer at a compression pressure of 98 kPa. (a) Photos depicting the contact and separation process over time of a 2-TENG composed of planar films, using a pushing tester (scale bars are 1 cm). i) Al makes full contact with the planar films in a downward direction (t=0 s). ii) As Al moves upwards, the uppermost planar film momentarily adheres to Al due to a sticky nature of the planar film (t=0.3 s). iii) After a brief period, Al and the planar film fully separate from each other with damping (t=0.6 s). (b) J_{sc} showing the damping phenomenon when the 2-TENG and planar film are in the separation process. (c) Adhesive strengths between the planar and microdome-patterned PDMS (M-PDMS) films at a compression pressure of 98 kPa, as measured by a texture analyzer.

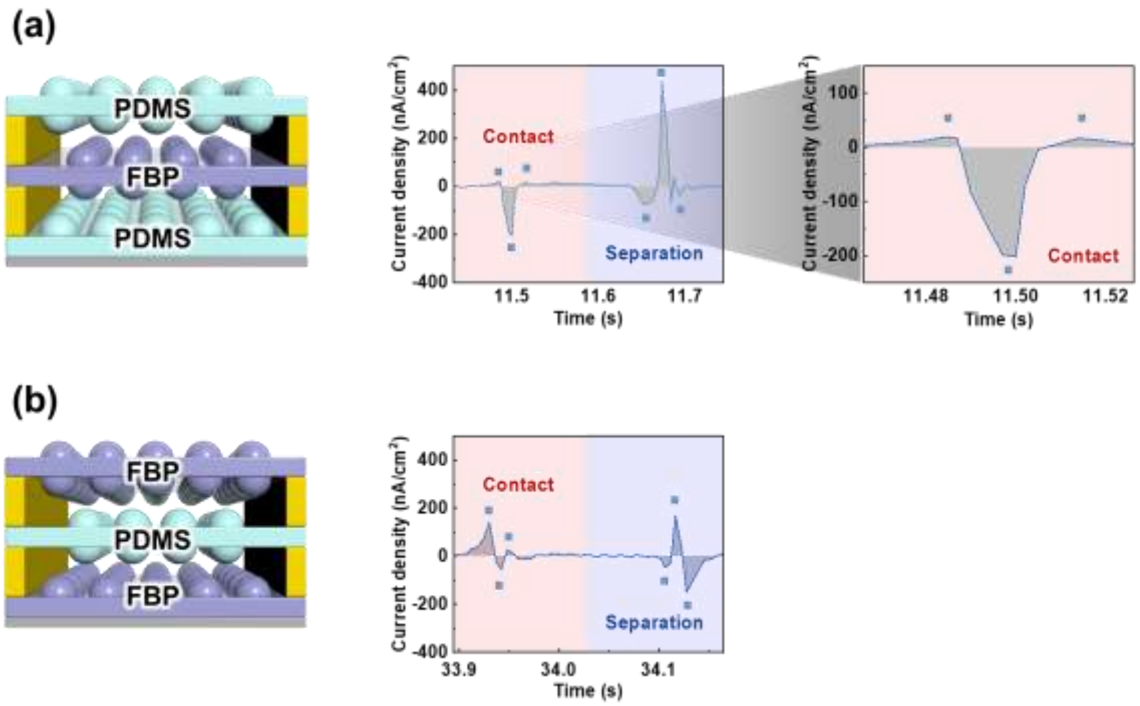

Figure S9. Comparison of J_{sc} between (a) 3-TENG with a PDMS–FBP–PDMS structure and (b) 3-TENG with an FBP–PDMS–FBP structure at an applied pressure of 9.8 kPa. The first contact layer is significantly important to generate distinct multiple spikes. Blue dots indicate the peaks of the multiple spikes.

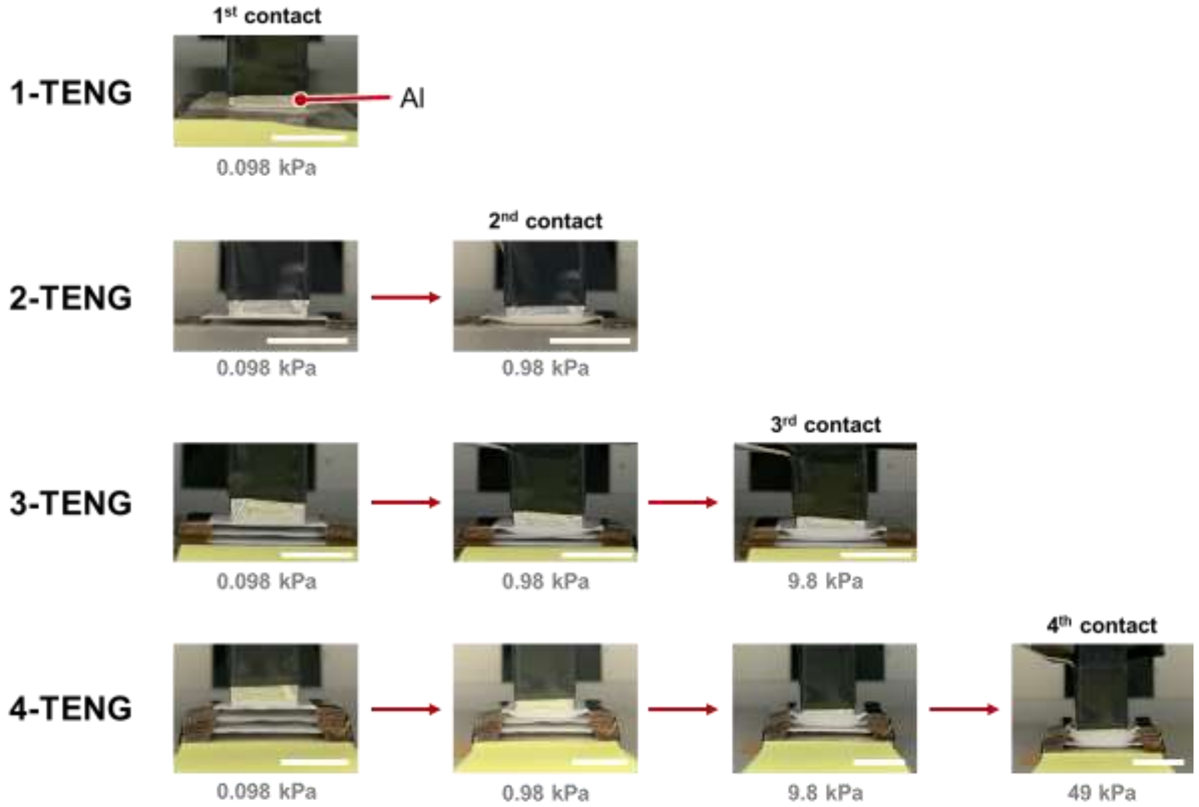

Figure S10. Digital images of M-TENGs under various applied pressures using a pushing tester. Controllable multiple spikes can be achieved through pressure-dependent, step-by-step contact. Scale bars are 1 cm.

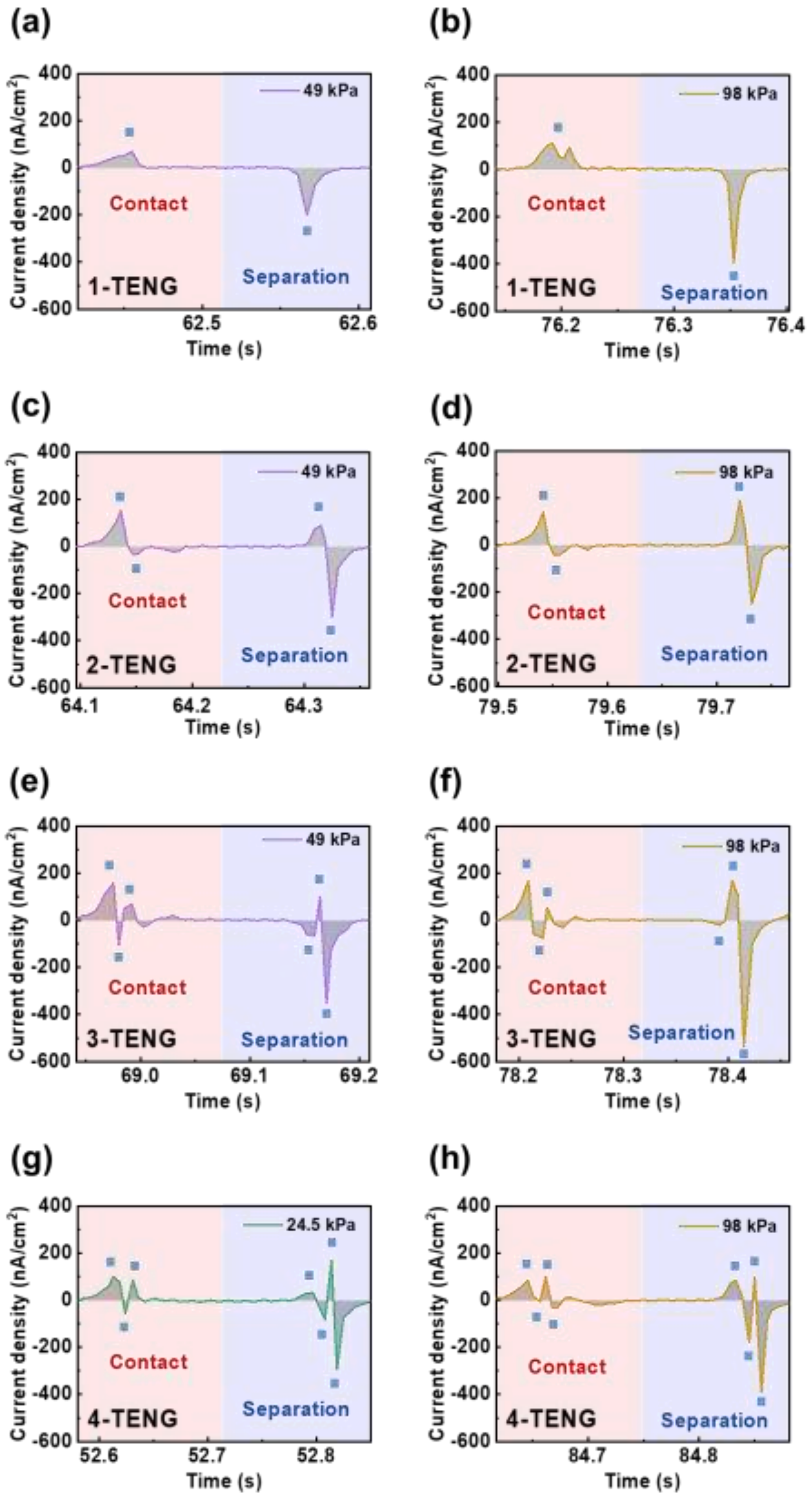

Figure S11. J_{sc} of (a, b) 1-TENG, (c, d) 2-TENG, (e, f) 3-TENG and (g, h) 4-TENG under various applied pressures. Blue dots indicate the peaks of the multiple spikes.

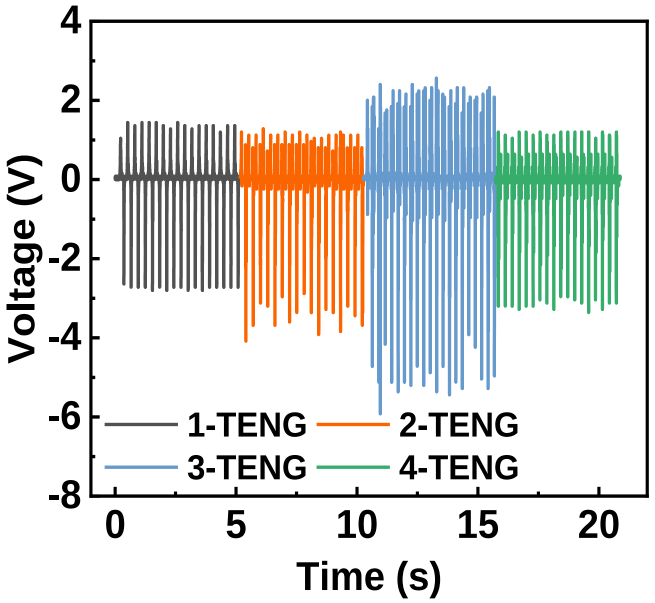

Figure S12. V_{oc} of 1-TENG, 2-TENG 3-TENG and 4-TENG at an applied pressure of 98 kPa. The V_{oc} of 3-TENG is much higher than that of others.

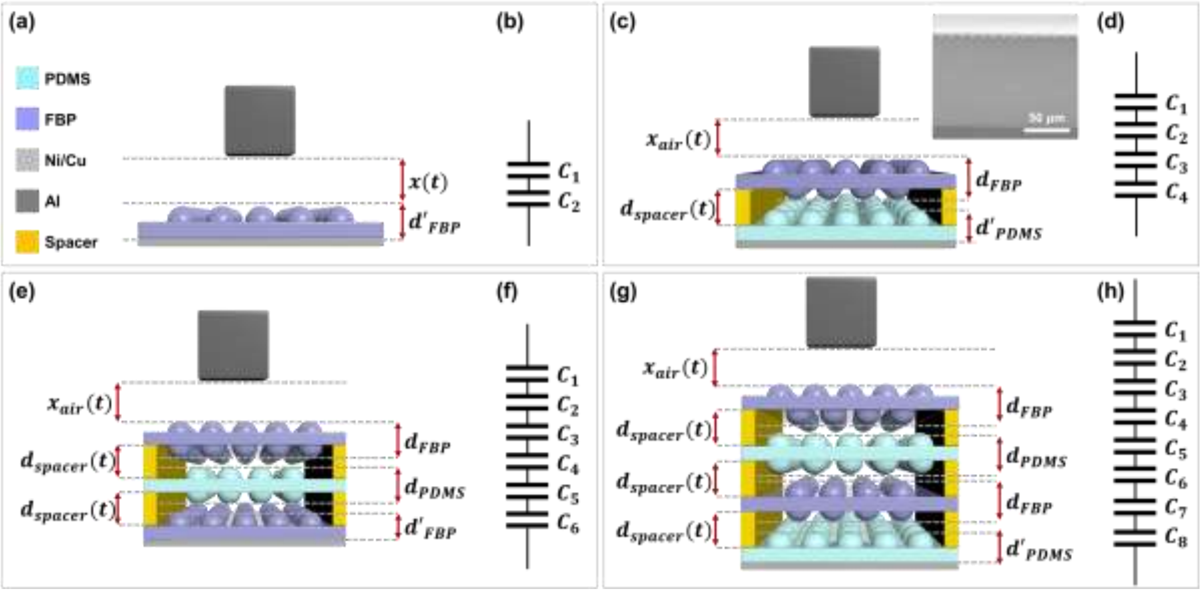

Figure S13. Schematic illustrations of theoretical models of M-TENGs and their equivalent circuits. (a, b) 1-TENG, (c, d) 2-TENG, (e, f) 3-TENG and (g, h) 4-TENG. The inset in (c) shows a cross-sectional SEM image of the microdome-patterned friction layer.

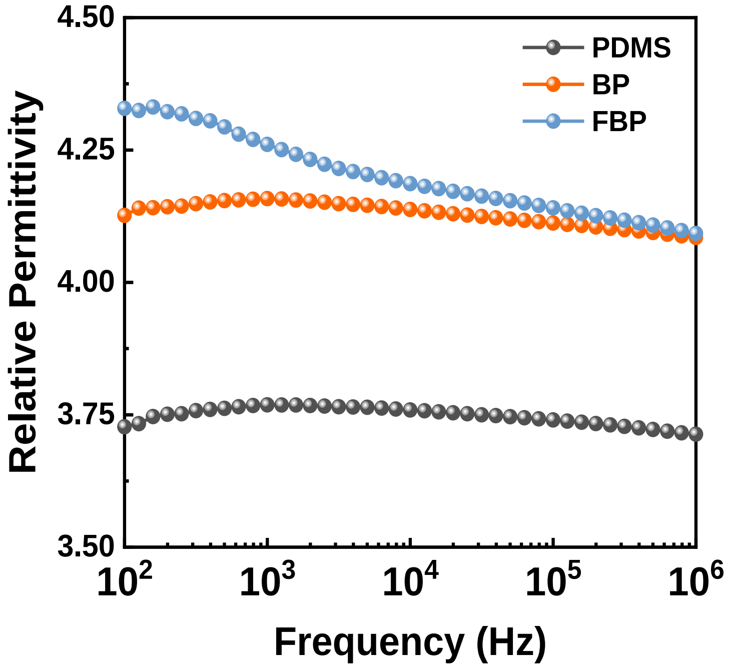

Figure S14. Relative permittivity of PDMS, BP (with 5 wt% BTO) and FBP, ranging from 100 Hz to 1 MHz.

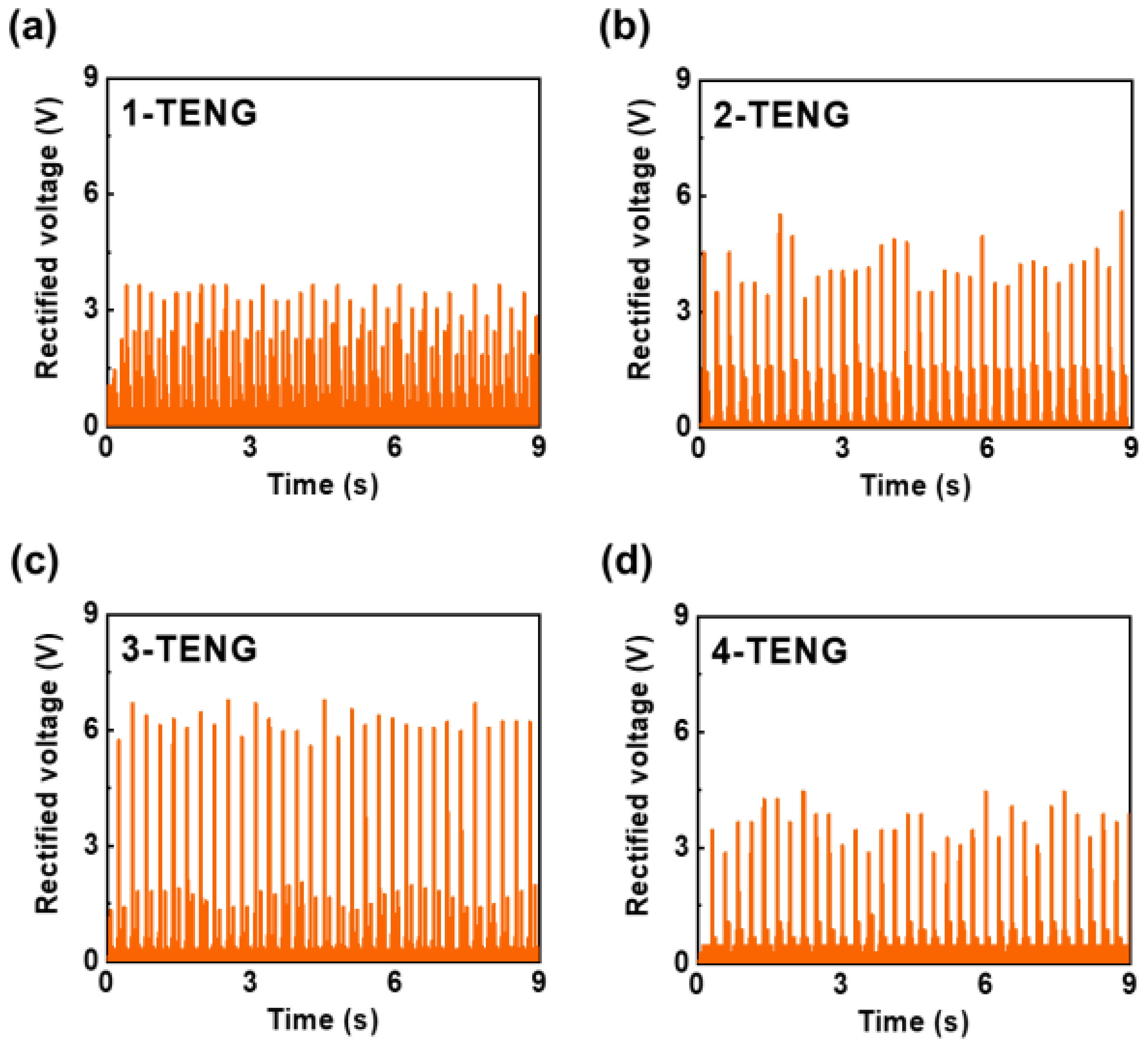

Figure S15. Rectified V_{oc} of (a) 1-TENG, (b) 2-TENG, (c) 3-TENG and (d) 4-TENG under an applied pressure of 98 kPa.

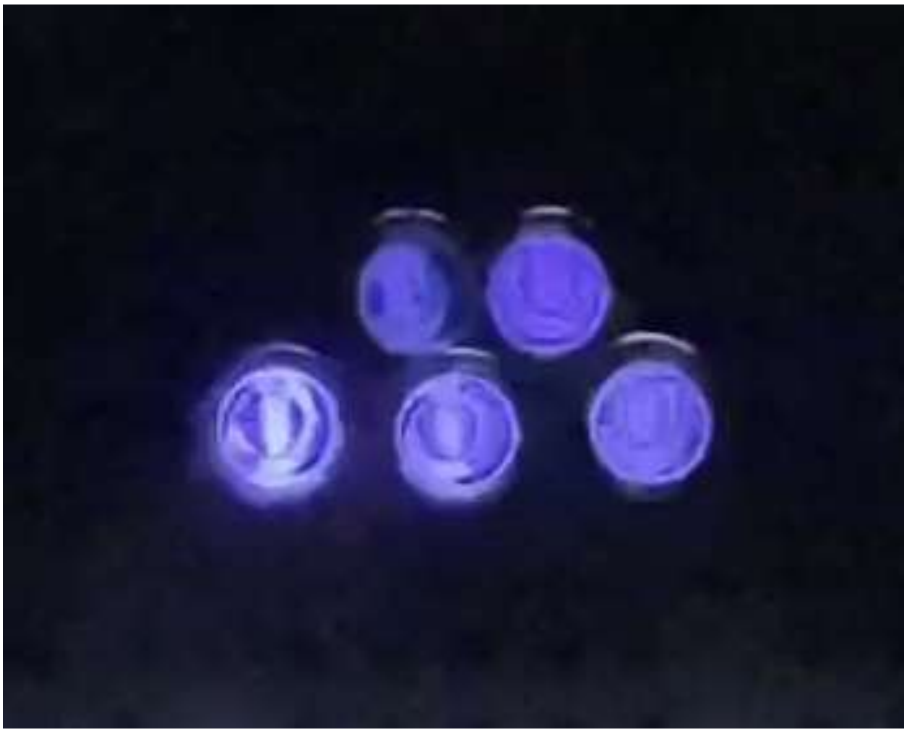

Figure S16. A digital photo of 5 blue LEDs operated by a 3-TENG with a full-wave bridge rectifier.

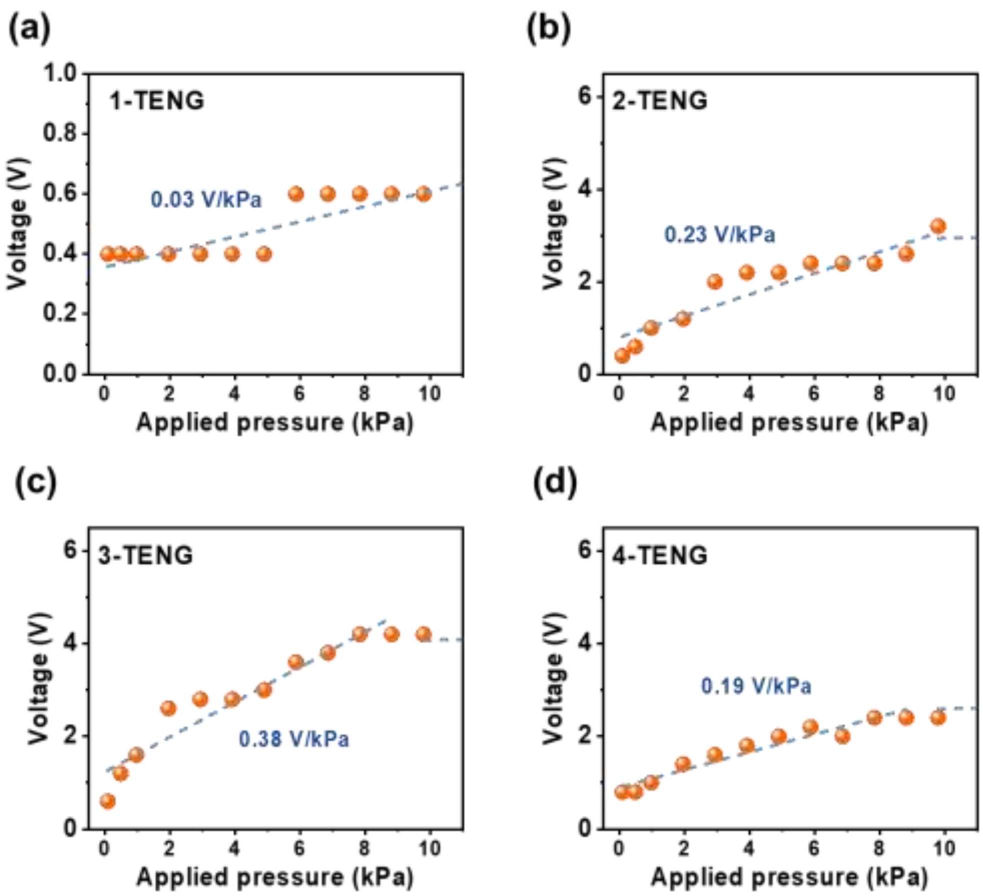

Figure S17. Magnified pressure-dependent output voltages and pressure sensitivities of M-TENGs at low-pressure range (0–9.8 kPa). At low pressure range, 1-TENG does not exhibit significant pressure sensitivity.

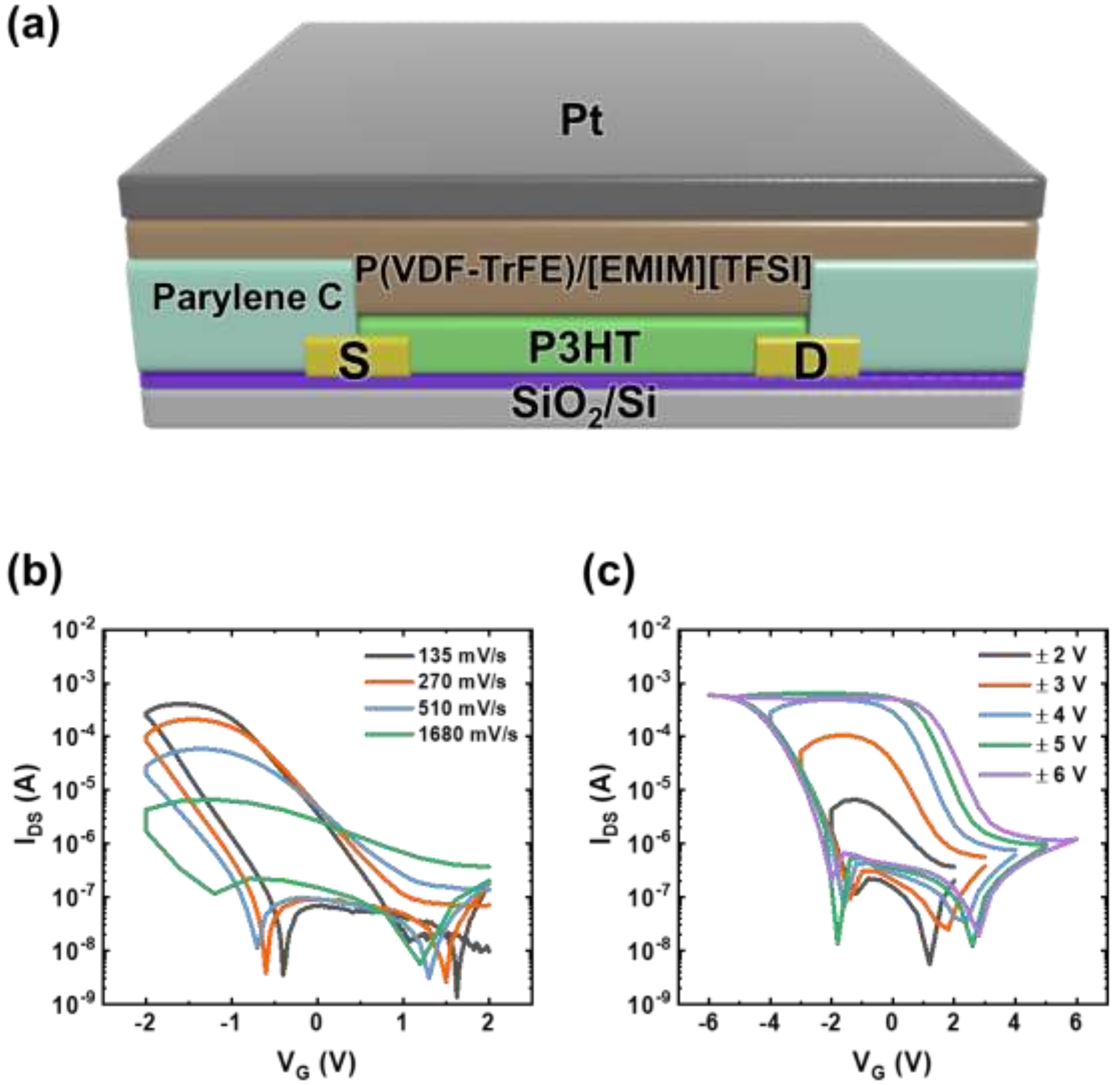

Figure S18. Characterization of the OECT. (a) Schematic illustration of the structure of the OECT. (b) Transfer characteristics (I_d-V_g) of V_g between 2 V and –2 V with various V_g sweep rates and (c) with different V_g sweep ranges (±2 V to ±6 V) at a sweep rate of -1680 mV s^{-1}. V_d was fixed at -0.05 V.

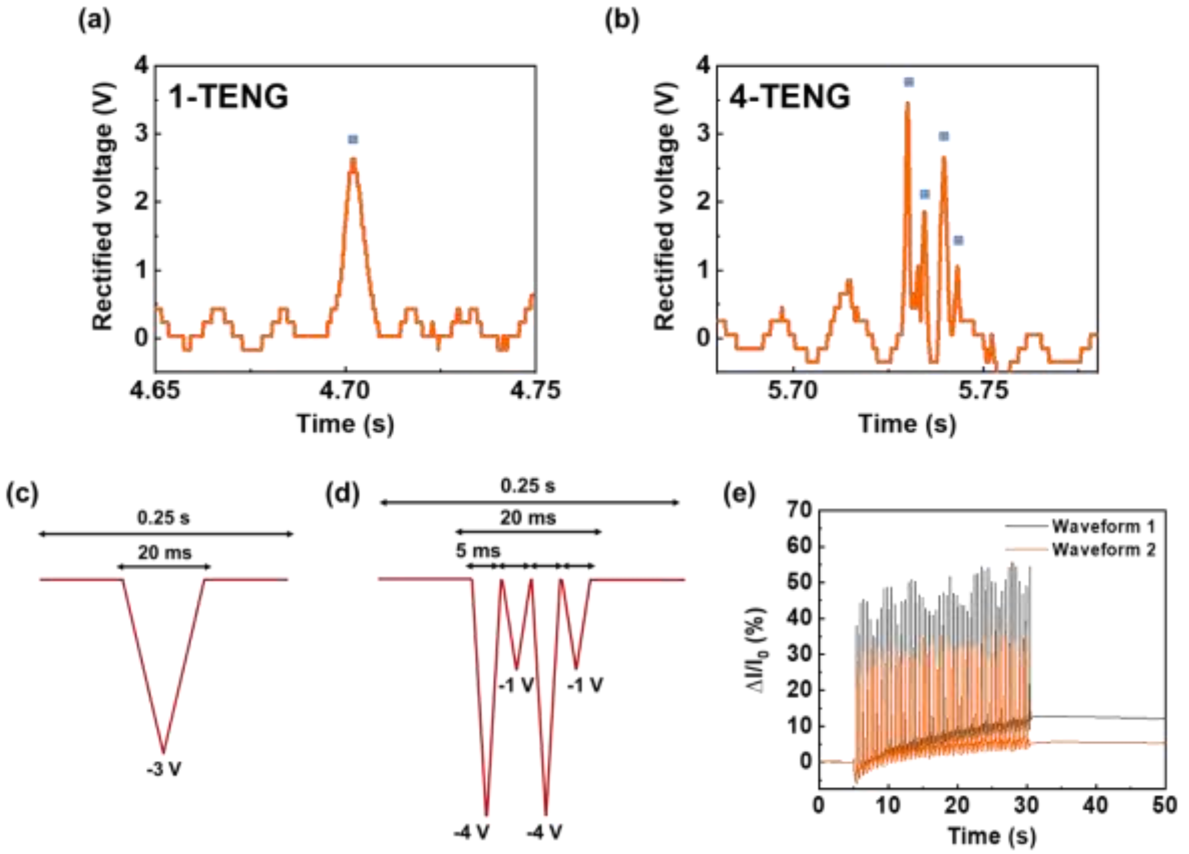

Figure S19. Magnified plot of the rectified voltage of (a) 1-TENG and (b) 4-TENG. Blue dots indicate the distinct peaks of multiple spikes. Simulated waveforms of (c) waveform 1 and (d) waveform 2, representing the rectified voltage of 1-TENG and 4-TENG, respectively, with a frequency of 2 Hz. (e) Drain current changes upon applying gate voltages using waveform 1 and waveform 2 (25 s at 2 Hz) *via* a function generator.

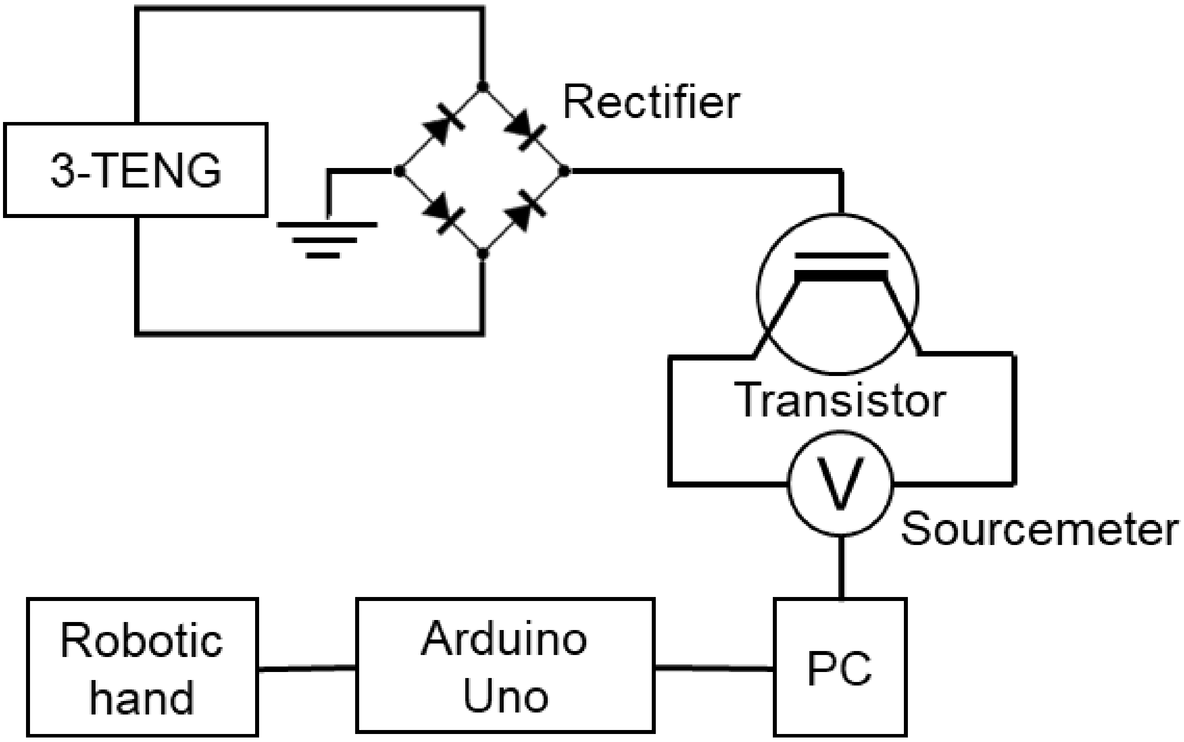

Figure S20. Circuit configuration for operating a robotic hand. The 3-TENG is connected to a rectifier to convert AC signals into DC signals. The DC signals are used for the gate voltage source for the OECT. The EPSC is recorded through a sourcemeter, and the robotic hand is operated by an Arduino Uno circuit.

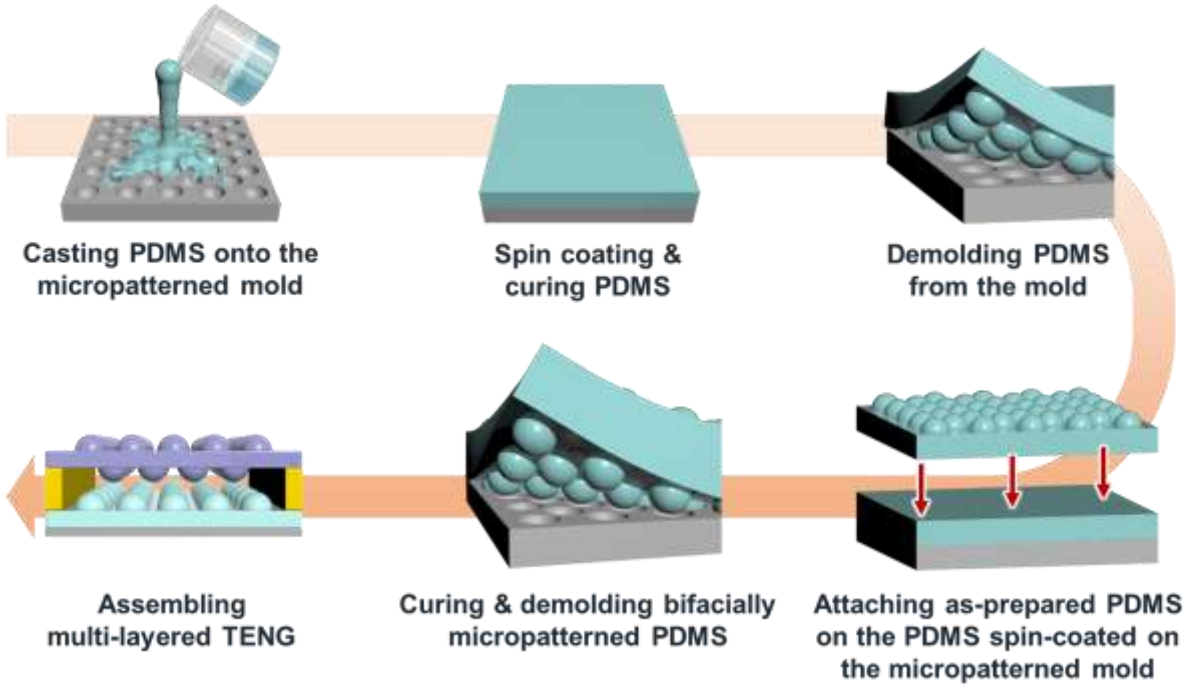

Figure S21. Schematic diagram of the fabrication method of the multi-layered micropatterned TENG.

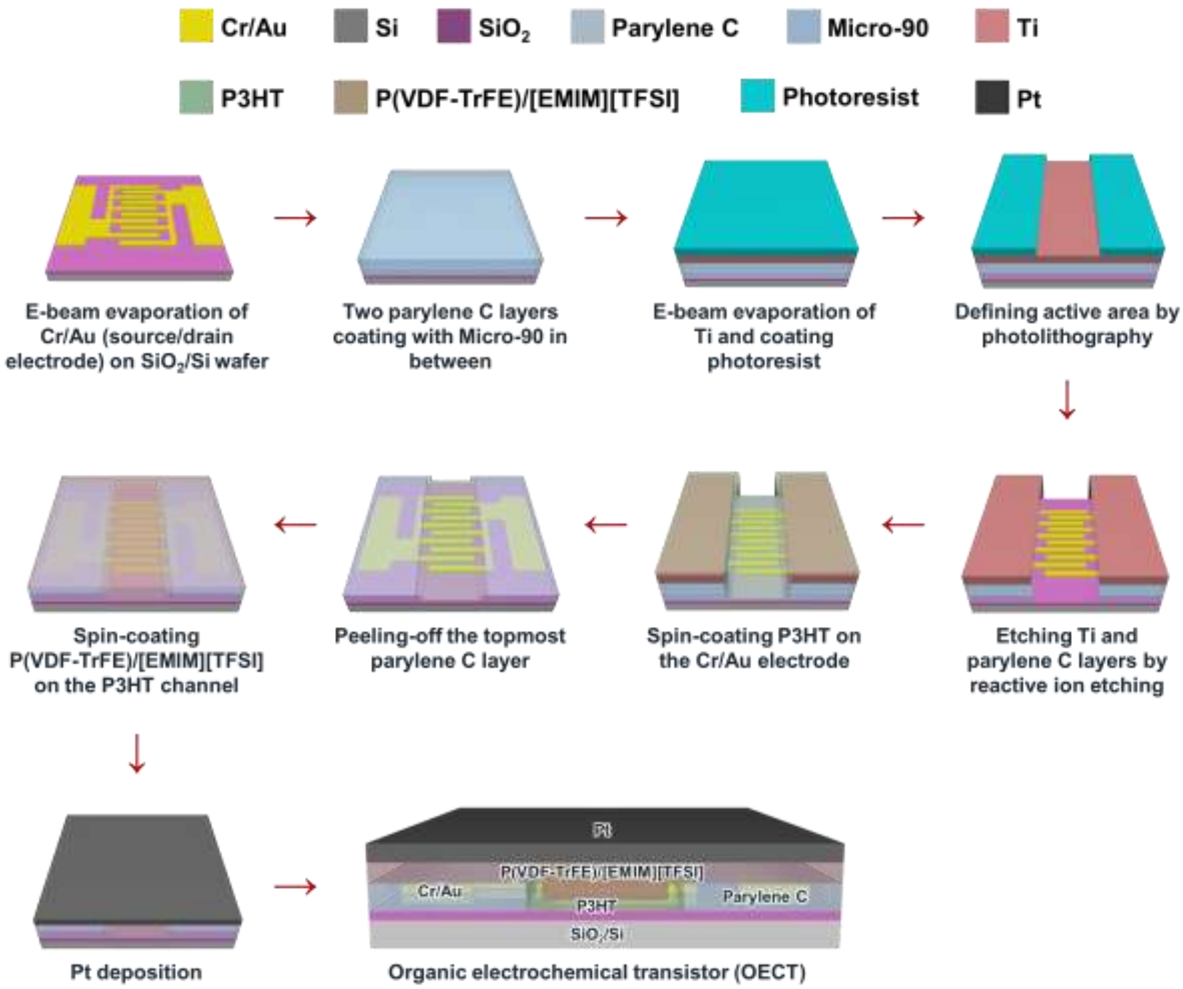

Figure S22. Schematic diagram of the fabrication method of the OECT.

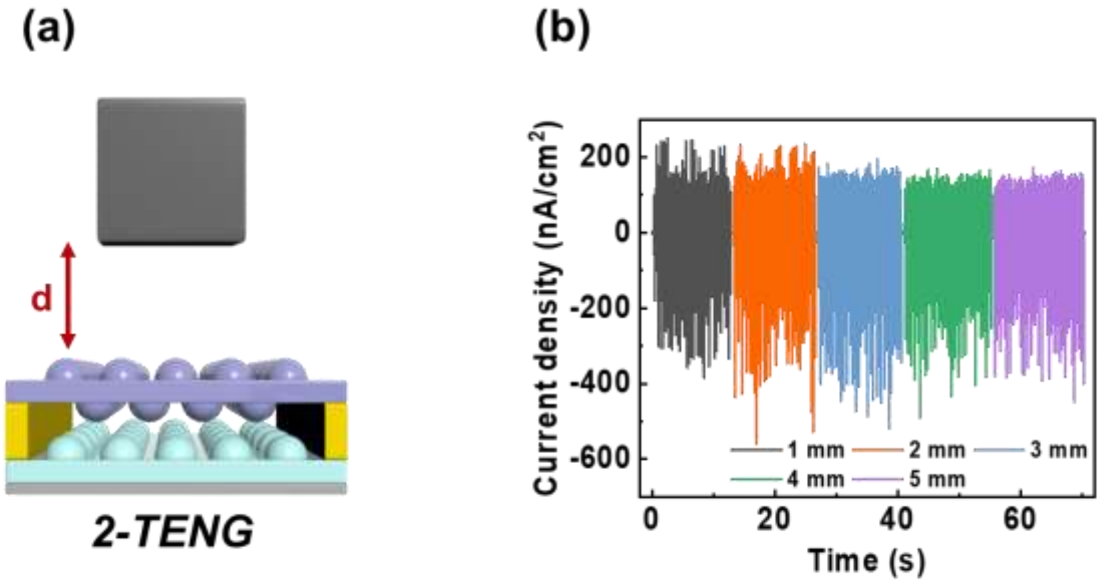

Figure S23. (a) Schematic illustration of a 2-TENG with a variable contact-separation distance. (b) J_{sc} of the 2-TENG with variation in contact-separation distance at an applied pressure of 98 kPa.

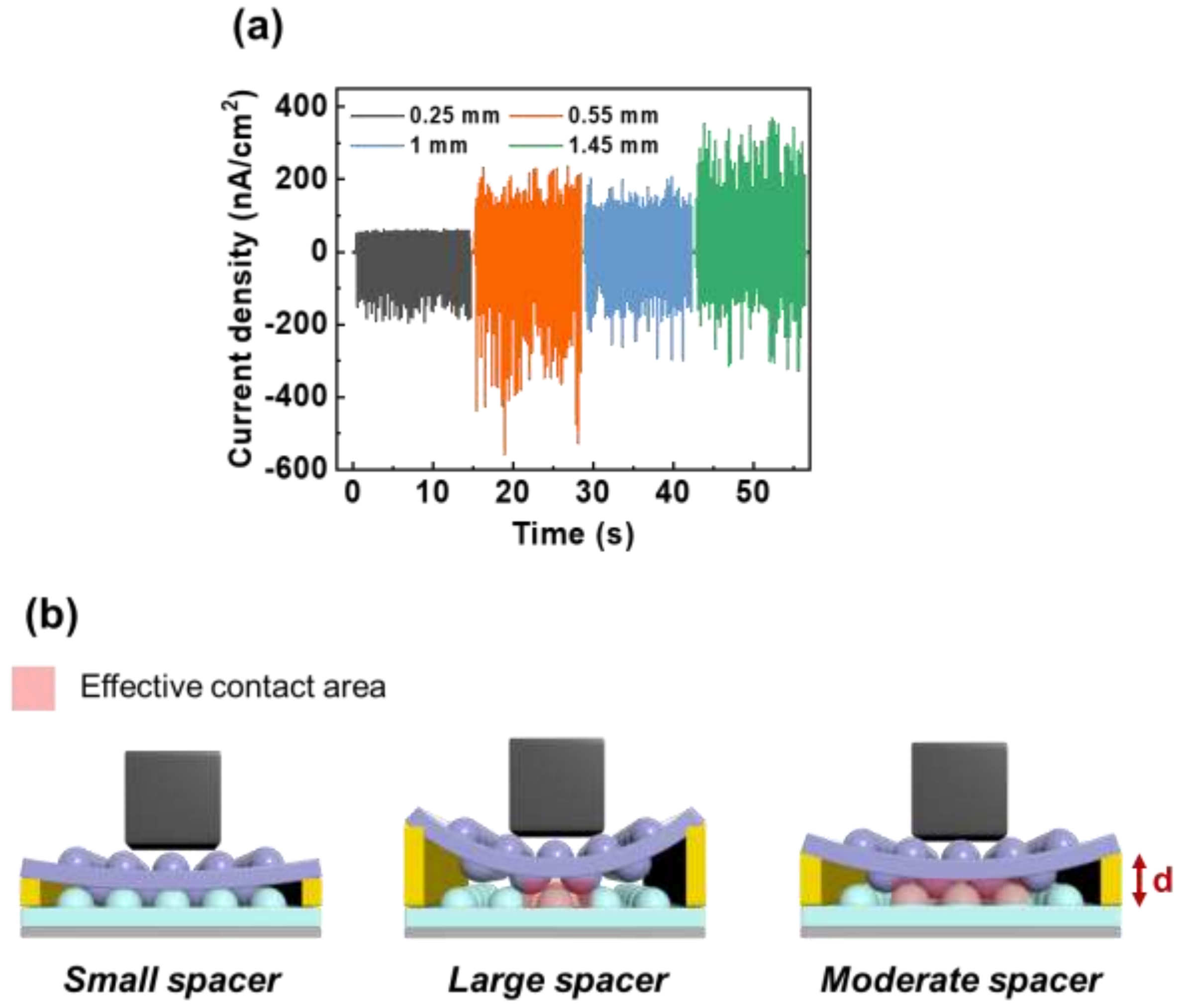

Figure S24. (a) J_{sc} of the 2-TENG with variation in spacer thickness at an applied pressure of 98 kPa. The optimum spacer thickness is 0.55 mm. (b) Schematic illustration showing different effective contact areas between the two friction layers depending on the spacer thickness. Choosing the optimal spacer thickness is critical for clear layer separation and large effective contact area.

Table S1. Comparison with the reported multi-layered TENG in previous literatures.

TENG material	Structure (contact area)	Output voltage [V]	Spike number	Application	References
PDMS–Au NPs/Al–Al	3-layered structure (4 cm^2)	300	6	Remote controller and the infrared sensor	[2]
FEP[a)]–Cu	Wavy structure (1 km^2)	250	2	Energy harvesting from water wave	[3]
Cu-PTFE[b)]-Al with corona charging	2-layered structure (49 cm^2)	2450	2	Self-powered LCD display system	[4]
Al–PVDF[c)]–Nylon or PU[d)]	3-layered structure (1 cm^2) or 7-layered structure (1.54 cm^2)	60	4 (Max=14)	Sound-wave-harvesting system	[5]
**Al – FBP – PDMS**	**3-layered structure (1 cm^2)**	**6.2**	**6 (Max= 8)**	**Artificial synaptic device**	**This Work**

[a)]FEP: fluorinated ethylene propylene; [b)]PTFE: polytetrafluoroethylene; [c)]PVDF: polyvinylidene fluoride; [d)]PU: polyurethane.

References

[1] J. J. Shao, W. Tang, T. Jiang, X. Y. Chen, L. Xu, B. D. Chen, T. Zhou, C. R. Deng, Z. L. Wang, *Nanoscale* **2017**, *9*, 9668.

[2] J. Chun, B. U. Ye, J. W. Lee, D. Choi, C.-Y. Kang, S.-W. Kim, Z. L. Wang, J. M. Baik, *Nat. Commun.* **2016**, *7*, 1.

[3] L. M. Zhang, C. B. Han, T. Jiang, T. Zhou, X. H. Li, C. Zhang, Z. L. Wang, *Nano Energy* **2016**, *22*, 87.

[4] T. Zhou, L. Zhang, F. Xue, W. Tang, C. Zhang, Z. L. Wang, *Nano Res.* **2016**, *9*, 1442.

[5] N. Cui, C. Dai, J. Liu, L. Gu, R. Ge, T. Du, Z. Wang, Y. Qin, *Energy Environ. Sci.* **2020**, *13*, 2069.
